# Non-Covalent Reactions Supporting Antiviral Development

**DOI:** 10.3390/molecules27249051

**Published:** 2022-12-19

**Authors:** Ilma Nugrahani, Emy Susanti, Tazkia Adawiyah, Safira Santosa, Agnesya Namira Laksana

**Affiliations:** School of Pharmacy, Bandung Institute of Technology, Bandung 40132, Indonesia

**Keywords:** multi-component system, antiviral, salt, cocrystal, physicochemical character, activity

## Abstract

Viruses are the current big enemy of the world’s healthcare systems. As the small infector causes various deadly diseases, from influenza and HIV to COVID-19, the virus continues to evolve from one type to its mutants. Therefore, the development of antivirals demands tremendous attention and resources for drug researchers around the world. Active pharmaceutical ingredients (API) development includes discovering new drug compounds and developing existing ones. However, to innovate a new antiviral takes a very long time to test its safety and effectiveness, from structure modeling to synthesis, and then requires various stages of clinical trials. Meanwhile, developing the existing API can be more efficient because it reduces many development stages. One approach in this effort is to modify the solid structures to improve their physicochemical properties and enhance their activity. This review discusses antiviral multicomponent systems under the research phase and has been marketed. The discussion includes the types of antivirals, their counterpart compound, screening, manufacturing methods, multicomponent systems yielded, characterization methods, physicochemical properties, and their effects on their pharmacological activities. It is hoped that the opportunities and challenges of solid antiviral drug modifications can be drawn in this review as important information for further antiviral development.

## 1. Introduction

Solid structure engineering growth is taking place in many areas, including pharmaceutical compound development with the revolution of solid analysis instrumentations revolution, especially the diffractometer [1,2,3]. Solid structure characters of drug compounds are determined by the bonding between atoms (intramolecular) and among molecules (intermolecular) [1,4]. On the other hand, the activity of a drug is primarily determined by its binding to receptors in the body. Therefore, the type of inter and intramolecular bonding become the central core of a drug’s performance, physicochemical properties, and activity aspects [5,6]. Furthermore, physicochemical characteristics of an active pharmaceutical ingredient (API), such as solubility [7,8,9], hygroscopicity [10], stability [11,12], and so on, may influence the onset and duration time of action of the drug in the body and assess its work. In addition, changes in the active site’s environment can also affect the drug’s potency. Hence, the solid structure alteration also can change the physicochemical performance and drug activity.

Several topics became the focus of discussion in drug solid structure studies, such as polymorphism, pseudopolymorphism or solvates/hydrates formation, and multicomponent systems arrangement through non-covalent bonds. A neutral or ionic interaction facilitates the multicomponent reaction of one API with another compound, producing cocrystal or salt. Those two reactions do not permanently change the drug’s chemical structure. Consequently, there are no changes in the binding with the receptor. In short, reversible interaction can improve drug performance without changing its activity.

Antiviral compounds have been developed along with the discovery of the virus itself. Dmitri Ivanovsky first discovered viruses in 1892, which attack a tobacco plant and cause a plantation disease. In 1898, the name “Virus” was introduced by Dutch biologist Martinus Beijerinck who found tobacco mosaic virus, which exhibited a small hollow rod shape and was formed by a single helical RNA strand and enclosed by a protein coat [13].

Timely, hundreds of multicomponent systems of API have been reported and patented, and many have been marketed. Data show that the most developed multicomponent systems are anti-inflammatory [14,15,16] and antipyretic agents [17,18,19], followed by antibiotics and anti-degenerative drugs. Nowadays, antiviral multicomponent development has increased with the intense spreading of viruses. An example of the marketed antiviral solid multicomponent system is lamivudine-zidovudine [20], which produces a synergetic effect on HIV. Next, the combination of adefovir dipivoxil-nicotinamide has also been patented to enhance antiviral activity against HBV [21].

**Figure 1:**
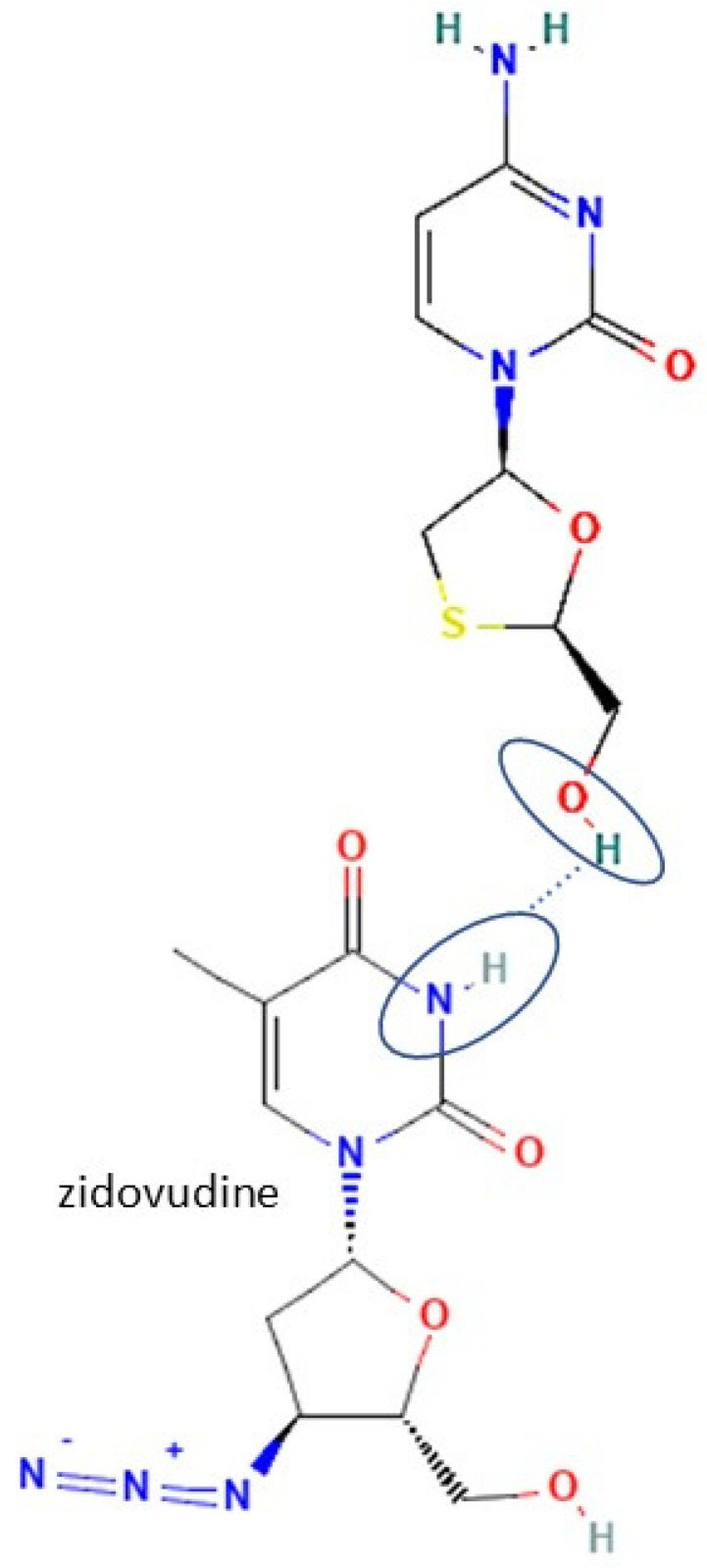
Multicomponent system antiviral lamivudine-zidovudine for HIV.

The multicomponent system formation is facilitated by intermolecular bonding between the different electronegativity functional groups. For example, Figure 1 depicts the “synthon” or interaction site, which is signed in the blue cycles, involving an amine site of zidovudine with a hydroxyl group of lamivudine [20].

Besides powder X-ray diffractometry, various microscopes, thermal analysis tools, vibrational spectrophotometers, and solid nuclear magnetic resonance also contribute to the new solid state phase innovations as the supporting instrumentations. Still, single crystal powder X-ray diffractometer has been proven to be the most reliable tool for determining the final structure until the atom and molecule position accurately in their space or three-dimensional conformation. In addition, in some cases, nuclear magnetic resonance is also helpful in supporting the structural study. The benefits from new solid phase preparation encourage further API development, especially their multicomponent systems preparation, which is expected to combine each component’s advantages.

Some methods have been utilized to obtain the appropriate multicomponent solid states, from conventional to newly updated plans. Conventionally, grinding, slurry, and evaporation methods have been broadly used, followed by extraction, microwaving [22], radiation, etc. Next, some processes have been reported to produce the preferred size of the particles at once, such as nano-cocrystal production [23]. Each method offers certain advantages. In this review, we also explain those methods to support integrative discussion step by step.

Hereafter, this review focuses on the antiviral solid multi-component system development, including the screening of the active pharmaceutical ingredient (API), the coformer/counterion, processing, characterization, structure determination, physicochemical properties change investigation, and the impact on their pharmacological activity. Finally, this article aims to comprehensively review and provide updated information from the last decade’s innovations in antiviral solid state development to support further development.

## 2. Antiviral

Antivirals are medications that are designed to help the human body to fight against certain viral infections. In 2004, there were almost 40 compounds that were officially approved that are capable of fighting against some types of viruses [24]. Then, in 2016 it was reported to be 90 antivirals that have been officially approved and patented [25]. The targeted viruses by these antivirals are influenza virus, human immunodeficiency virus (HIV), hepatitis B virus (HBV), herpes simplex virus (HSV), respiratory syncytial virus (RSV), varicella-zoster virus (VZV), and cytomegalovirus (CMV), and hepatitis C virus (HCV) infections [24].

Diseases that are caused by the virus are plenty. Examples of the viral disease include influenza, which causes some severe aching that comes along with fever; severe acute respiratory syndrome; chickenpox which is caused by the herpes zoster virus that makes the person will have some itchy inflamed blisters; herpes, caused by herpes simplex or herpes zoster, which comes with the eruption of the small blister-like vesicles; hepatitis which is characterized by liver inflammation; cold sores; measles is the disease-causing fever and a red rash on the skin that often infects children; shingles that come with the painful inflammation of the nerve ganglia, with a skin eruption; poliomyelitis is the disease that causes temporary or permanent paralysis; acquired immunodeficiency syndrome (AIDS) that caused by HIV that is often causing several symptoms such as shortness of breath, diarrhea, white spots or unusual blemishes in and around the mouth, fever, vision loss, and other pneumonia-like symptoms; smallpox; etc. [26].

Based on how the antiviral works, there are two different interventions for designing the antiviral drugs: targeting the host factors or the virus. Direct virus-targeting drugs inhibit virulency, including attachment, uncoating, entry, polymerase, protease, integrase, nucleoside, nucleotide reverse transcriptase inhibitors, and non-nuclide reverse-transcriptase inhibitors [26]. Meanwhile, protease inhibitors (darunavir, atazanavir, and ritonavir), viral DNA polymerase inhibitors (acyclovir, valacyclovir, valganciclovir, and tenofovir), and an integrase inhibitor (raltegravir) are included in the list of “Top 200 Drugs” by sales for the 2010s [25]. In addition, those host-direct antiviral factors control the unprecedented viral infection spread [27]. However, during clinical investigations, we should consider the probability of cellular side effects or cytotoxicity on host cells [26].

**Figure 2:**
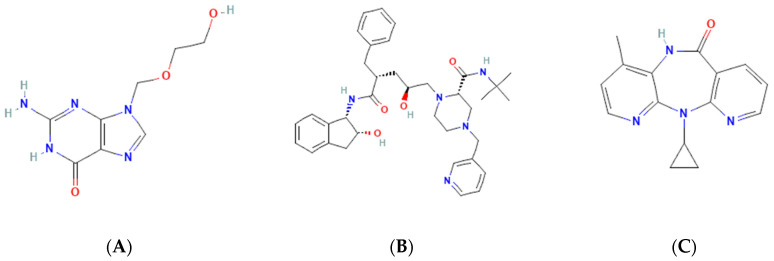
Molecular structure of (**A**) acyclovir [29], (**B**) indinavir [30], and (**C**) nevirapine [31].

Most antiviral drugs are highly water soluble and have high molecular weights (>200 g/mol). However, some of them, such as acyclovir, indinavir, and nevirapine, have low water solubility. The high solubility of antiviral is caused by the carbonyl oxygen of the ketone group’s ability to form hydrogen bonds with the water [25], which the structure of acyclovir, indinavir, and nevirapine are shown in Figure 2. Previous studies have shown that tannins with a higher molecular weight typically have more potent antiviral activity. In this case, the extracts and tannic acid (1702 g/mol) can inhibit both IAV receptor binding and neuraminidase activity. Meanwhile, gallic acid (<500 g/mol) only inhibits neuraminidase [28]. The summary of antiviral’s work mechanisms and indications can be seen in Table 1.

## 3. Solid State Engineering

Solid state engineering is a strategy to improve the physicochemical properties of compounds such as API without changing structure and activity. Multicomponent system composing is one of the solid state engineering processes and is defined as a phase that contains one or more components in a fixed stoichiometric ratio. The compound interaction can involve intermolecular or intramolecular bonds, such as hydrogen, ionic, and covalent bonds. Each multicomponent product with the same compound has a different property since it has a different composition. The multicomponent system is divided into salt (ionic interaction), pseudopolymorphism (hydrate/solvate formation), and cocrystal (neutral interaction).

### 3.1. Interactions Supporting Solid Engineering Approaches to Pharmaceutical Development

Many drugs have polymorphism and pseudopolymorphism. A compound’s ability to crystallize into more than one form with different arrangements of the molecules in the solid state but identical in terms of chemical content is a definition of polymorphism. The differences are the arrangement and their conformations because each crystal packing involves a certain lattice energy [32]. Thermal analysis can detect or differentiate each polymorph by measuring the melting point [33]. The higher melting point indicated a higher energy bond since it needs higher energy or heat, which degrades the bond between the compound and atom. Meanwhile, pseudopolymorphism is a crystal system that contains solvent molecules. It can be classified into solvate and hydrate. Solvate consists of ions or molecules of solute with surrounding solvent besides water [34]. Solvate can exhibit polymorphism by different arrangements of their constituent molecules in the structure [32]. Next, a hydrate is a form that consists of one or more water molecules inside the system. Therefore, it can also exhibit polymorphism by different water molecule amounts in the structure.

Polymorphism and pseudopolymorphism can be obtained by methods such as sublimation, crystallization, evaporation from a binary mixture of solvents, vapor diffusion, thermal treatment, desolvation, precipitation, and grinding [35]. Most solvate forms are yielded by the crystallization method. The pseudopolymorph preparation was challenging, especially in the screening process, since the molecules have high conformational flexibility. A high degree of conformational flexibility affected the polymorph product, and the outcome was primarily out of the prediction [35,36]. Every polymorph has different properties since they have varied arrangements. For example, Adefovir is an antiviral with four different polymorphs; every polymorph has a different thermal and solubility profile [37]. The adefovir development uses the polymorphism method purposes to find a soluble form.

Next is salt, a solid compound formed by two or more ionizable components with a negative or positive charge. It also involves the transfer of hydrogen atoms between acidic and basic functional groups. Salt formation is a suitable strategy for preparing effective and safe dosage forms of various drugs. Since it is an ionizable compound, it will lead to the high solubility of a drug [38]. Besides, salt formation has been developing to improve other physicochemical properties, dissolution, stability, altered gastrointestinal absorption, and antibiotic potency [39]. One salt multicomponent reported is arbidol antiviral, which, combined with benzoate and salicylate anions, improves thermodynamic stability and solubility [40].

Afterward, a neutral multicomponent system/cocrystal is a solid structure consisting of two or more compounds that form a crystal lattice in one phase supported by non-ionic intermolecular bonding. It was first discovered in 1844 and was characterized in 1958, but the term “cocrystal” was first used in 1963 by Lawton and Lopez [41]. This multicomponent system can be classified into two major groups, namely molecular and ionic cocrystal. As it is named, a molecular cocrystal consists of two or more different neutral compounds arranged by hydrogen or halogen bonds.

Meanwhile, an ionic cocrystal consists of at least one ionic compound supported by charge-assisted hydrogen bonds [39,42]. Most of the cocrystal product was formed by hydrogen bonds, which caused solubility improvement. Hydrogen bonds are more attracted to the water molecule and solve the cocrystal form even if it has a compound that does not ionize efficiently [43]. Cocrystal formation is currently used to gain physicochemical properties improvement without changing the therapeutic activity [37,41]. Cocrystal products can maintain therapeutical activity because there are no covalent bond changes or damages. It only involves hydrogen bond changes [44].

Different from salt, ionizable and non-ionizable compounds can form cocrystals. Cocrystal formation can be predicted by ΔpKa calculation. It can be produced if the ΔpKa value is 0 < ΔpKa < 3.75. In this rate, the chance of hydrogen bond formation is high. On the other hand, if the ΔpKa value is > 3.75, it will produce a salt form since it leads to electron transfer. Meanwhile, if the ΔpKa value is in rate 3–5, the product will be in the acid form [45,46].

Based on its charge, the cocrystal can be classified as; a zwitterionic cocrystal formed by hydrogen bonds interaction between drug and zwitterionic coformer. First, this zwitterionic compound may lead to the formation of charge-assisted hydrogen bonds [11,45]. Next, salt cocrystals are produced by intermolecular interaction influenced by ΔpKa differences between used coformer, both acid and base compounds [47]. Afterward, a base cocrystal may be formed by coordination between the Schiff base and metal atom in the coformer, which involves the metal coordination bonds [48]. Last, an acid cocrystal is obtained by interacting an acid compound with a carbonyl or an aromatic nitrogen group [49].

Multicomponent systems can be created by combining the API with a suitable compound, namely coformer. It must be non-toxic and classified as a GRAS (generally regarded as safe) compound. Coformer selection can be made by synthon theory and solubility theory. Synthon theory states that the coformer should complement API’s functional group. The multicomponent system involves supramolecular synthons responsible for binding and structure arrangement. It is divided into two kinds. First is homosynthons, a supramolecular consisting of the same functional group; second, heterosynthons consists of the different active group [37]. On the other hand, solubility theory states that API and coformer should have a different polarity, which the combination product can improve the solubility and stability [50]. Based on the coformer used, cocrystals can be produced by many kinds of combinations, such as drug-drug [51,52], drug-nutrient [52,53], drug-vitamin [54], drug-excipient [55,56], and drug-solvent [57].

### 3.2. Multicomponent Systems Preparation Methods

Several methods can be applied to produce the multicomponent compound: solvent-based, grinding, and heating. The solvent-based methods use a large amount of solvent, an organic or a combination of organic solvent with water. The solvent method is divided into slow evaporation, fast evaporation, cooling reaction, spray drying, and freeze-drying. The evaporation method involves nucleation and cocrystal growth while the drug and coformer are entirely dissolved in solvent until saturated. In this condition, API and coformer completely interact, and supersaturation may occur. When the solution is supersaturated, solvent removal will occur through evaporation. While the solvent is evaporated, the molecule in the solution binds with hydrogen bonds [9].

The evaporation process may be conducted slowly or fast. It depends on the using chamber. For slow evaporation, the solution of the drug and coformer is transferred into a chamber that has a smaller air surface, which makes the evaporation goes slowly since the contact of air and solvent is limited [58]. This method is generally used to produce a single crystal multicomponent, and the slow evaporation provides a bigger size of crystal product [59]. Meanwhile, the fast evaporation method uses a chamber with a broader air surface, which provides a bigger surface for solvent and air to interact. The more rapid evaporation makes the obtained crystal have a smaller shape because the kinetic energy in the solution is more extensive, and crystals tend to close by [37]. The advantage of the evaporation method is producing a thermodynamically stable crystal, but it needs a large amount of solvent.

Next is the cooling method; the process is purposed to prepare large-scale and purified crystals by involving a relatively bigger mass with temperature control. In this method, a mixture of drug and coformer is dissolved in a thermodynamically stable solvent until saturated. And then, the crystallization at a cool temperature (0–10 °C) to gain the supersaturated condition. Then, a spontaneous process produces the crystals. As a result, most cooling crystallization products have uniform particle size distribution [60,61]. Finally, the cooling method also can be conducted using nitrogen gas, named the “freeze-drying” method, by applying the unsaturated solution into a nozzle supported by pressure control [62]. This process removes the unsaturated solution consisting of drug and coformer and makes the other part highly saturated. Finally, it freezes the solution and reduces the pressure to sublime the water directly from the solid phase to the gas phase [63].

On the other hand, the grinding method is divided into two main kinds. First is neat grinding, which mixes the drug and coformer with pressure, while the grinding process uses mortar without any solvent involved [64]. This method produces a cocrystal in large amounts faster because there is no time to wait for the evaporation process. Still, the homogenous of this method is relatively poor in large-scale production [65]. Second is solvent-assisted grinding, which adds a small amount of solvent to a grinding process. The solvent acts as a catalyst without waste production [66]. This method is commonly mentioned as greener than evaporation since it only needs a small amount of solvent [67]. However, the produced multicomponent is less stable than the neat method product [68].

Meanwhile, the heating method involves heat in the preparation process, i.e., hot melt extrusion, isothermal slurry, and microwave methods. Hot melt extrusion uses heat and pressure to melt the drug and coformer in an extruder. The mixing and interaction between the drug and excipient happen while both are melting [6]. This method’s advantages are decreasing time and chemical waste since it does not need any solvent [60]. However, this method can only be used for thermostable compounds [68]. Next is the isothermal slurry method. This method is conducted by suspending the drug and coformer mixture. Again, water can be used as a solvent, and the pharmaceutical and coformer are not to be fully dissolved. But in this method, there is a heating process to facilitate the reaction [60]. The last is microwaving method using. First, the drug and conformer were mixed by grinding in the mortar, but in a shorter time, they were heated using a microwave [22]. Again, this method reduces the grinding time and increases the amount of the reaction product [65].

### 3.3. Multicomponent System Characterization

After the multicomponent sample was obtained using various methods, the samples were characterized using different Instruments. First is thermal analysis, which can analyze the thermal profile of the sample. It also can be applied to sample identification by measuring the specific melting point, purity confirmation, and molecular mass of solid state determination [69]. Thermal analysis can be done using various instruments such as semimanual electrothermal, differential scanning calorimetry (DSC), differential thermal analysis (DTA), and thermogravimetry (TGA). Semimanual electrothermal is an instrument that uses electricity as a heat source to heat the sample inside the capillary tube in the sample holder. The capillary tube used in semimanual electrothermal is a side-closed capillary tube. The sample is inserted into the capillary tube at about 2–3 mm [70]. The physical changes caused by heating can be observed on the holder until the melting point is reached and recorded [71].

Next is differential scanning calorimetry (DSC). This instrument can analyze the thermal profile by measuring the heat flow over a temperature in the sample holder of the DSC instrument consisting of two parts of placement: reference and sample [70]. An aluminum plate is commonly used to reference and covers the sample. The reference is needed to compare the empty plate weight with the sample plate so that the calculation of the heat flow changes is exact. The DSC measurement results in a thermogram graph containing thermal information of the sample by Y axis is heat flow (mW), and X axis is Temperature (°C). The endothermic peak is shown by a descending curve indicating dehydration, melting, and degradation. Meanwhile, the rising peak is a crystallization point [72]. Thermal measurement using DSC has been used for characterized multicomponent products such as adefovir-dipivoxil with dicarboxylic acid [71]. This measurement can also identify the new phase, as reported in [59].

**Figure 3:**
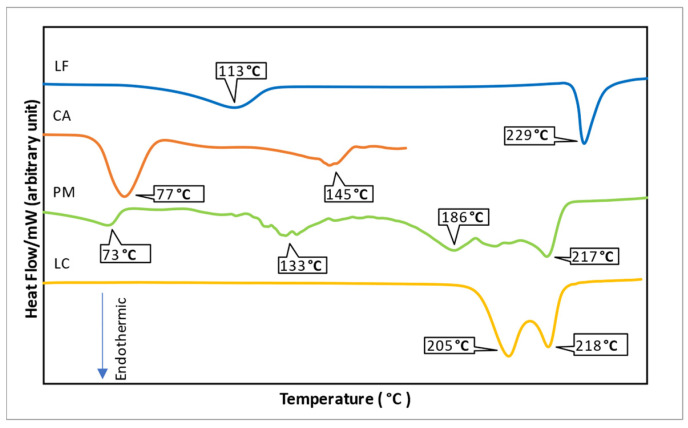
DSC thermogram of levofloxacin-citric acid [59].

For example, Figure 3 shows the DSC thermogram of the levofloxacin-citric acid multicomponent, which is different from its parent drug, indicated by other melting points and dehydration peaks. The parent drugs show water molecules release represented by an endothermic peak at a range (70–100 °C). Meanwhile, the multicomponent product does not have an endothermic peak at that range, indicating that they have different hydrate profiles. On the other hand, the multicomponent melting point showed by the endothermic peaks at 205 and 218 °C, lower than the single component, which melted at about 300 °C [59]. This different thermal profile confirmed that the multicomponent product is a new phase.

**Figure 4:**
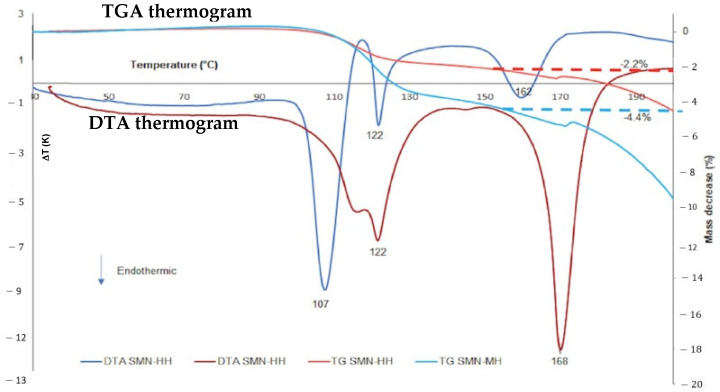
Thermogravimetry (TGA) and differential thermal analysis (DTA) of sodium mefenamic nicotinamide hemihydrate and monohydrate. Reprinted with permission from ref. [9]. Copyright 2021 Elsevier.

Besides DSC, differential thermal analysis (DTA) and thermogravimetry (TGA) have also been used widely for thermal analysis. Both methods are usually compiled in a measurement. DTA measures the heat differences between reference and sample over temperature increase. The result of DTA measurement is a thermogram graph which consists of delta temperature (K) as Y axis and temperature (°C) as X axis [23]. The advantages of DTA measurement are high sensitivity and can be used in super high temperatures, but it can’t determine total calory energy. For example, a DTA-compiled TGA thermogram can be seen in Figure 4. First, the DTA thermogram shows the melting point of sodium mefenamate nicotinamide hemihydrate (SMN-MH), and monohydrate (SMN-HH) found at 162 °C and 168 °C, respectively, indicated by the endothermic peak [9].

Next, TGA measures a sample’s weight changes over a temperature increase. Its thermogram was obtained by plotting the mass changes (%) on the Y axis and temperature (°C) on the X axis [51]. Analysis using the TGA instrument provides information about physical phenomena, including phase transition, absorption, and desorption. The TGA thermogram in Figure 4 indicates the mass changes at 155 °C. The mass of SMN-MH decreased by 2.2%, representing half the water molecule (hemihydrate) released after that point. Meanwhile, the mass of SMN-HH decreased by 4.4%, equal to one water molecule (monohydrate) [9].

Not only to characterize the obtained multicomponent, but the thermal analysis is also commonly used in a screening process to determine the molar ratio of each compound. For example, Ferreira et al. made a binary phase diagram of riboflavin and norfloxacin in several molar ratios [73]. A binary phase diagram pattern indicates a multi-component system formation. The appropriate molar ratio showed the highest melting point between the two lowest melting points [10].

The multicomponent system is then prepared based on the fixed molar ratio screened and observed using a binocular microscope to recognize the shape of the new solid structure product conventionally. The form of the obtained multicomponent can be needle-like [74], rod shape [75], transparent square [76], etc. Next, the new phase formation is confirmed by characterized using powder x-ray diffractometry (PXRD). The multicomponent, constituent compounds and their physical mixture are analyzed and compared to verify the new phase formation. The physical mixture diffractogram showed the combination peaks indicating no interaction and is not a new phase [77,78]. If the reaction is incomplete, various distinctive and constituent compound’s peaks still exist [62].

After the solid state characterization, vibrational spectrophotometry structurally identifies the new interaction in the multicomponent system. Vibrational spectroscopy can detect the exchange in the multicomponent by evaluating the interaction between the molecule and infrared lighting from the radiation source of the instrument. This analysis uses a relatively low energy level compared to photo-spectrometry, in which the wave energy used can interact with almost all electron bonds. The commonly used vibrational spectroscopy are Fourier transforms infrared, Raman, and terahertz spectroscopy [78,79,80,81]. Fourier transform infrared spectroscopy (FTIR) is a non-destructive analytical-qualitative method that can identify molecular interaction by detecting the existing bonds [64]. The FTIR spectrum of the new interaction and the constituent compounds can be compared to confirm the multicomponent system formation. For example, the changes in broadband of the spectra at wavenumber about 3500 cm^−1^ indicated a shift in water molecule number [82]. Meanwhile, the expected bonds that may occur in multicomponent systems are C=O, O-H, and N-H, which showed by the appearing band at wavenumber between 1680–750 cm^−1^ [83].

Like FTIR, Raman spectroscopy is an instrument that uses vibrational energy to detect the interaction. Besides vibrational, this instrument also applies rotational and low-frequency modes of the molecules. Differing from FTIR, Raman monochromatic visible light to near-infrared is used as the light source. They are utilized to get information related to fingerprints [84]. The monochromatic radiation is passed through the sample and may get reflected, absorbed, or scattered [85]. The advantage of Raman is the sample can be in a solid, liquid, or gas phase [84]. It has been stated that the multicomponent formation has been confirmed using this method, which is indicated by shifted bands to lower or higher wavenumber and appearing of new bands in the multicomponent spectrum [84]. Meanwhile, Raman spectroscopy can compare the intermolecular and intramolecular energy shown by the frequency shifting [85]. This tool may determine the polymorph and multicomponent based on their Rayleigh, Stokes, and anti-Stokes scattering. For example, the hydrate formation can be detected in the 3750–3250 cm^−1^ range. Meanwhile, the new interaction of the other functional groups, such as COO- and NH-, can be read in 2000–1500 and 3500–3250 Raman shifts (cm^−1^), respectively.

In addition, Terahertz (THz) spectroscopy also observes the molecular bonds of multicomponent [86]. Compared with analysis using infrared waves and visible light, the development of THz is quite unpopular because it is challenging to find the radiation source and detector of this radio electromagnetic (REM). THz’s wavelength is between infrared and optical, 0.1–10.0 THz/300 µm—30 mm, and may be used to observe the physical and chemical properties by showing fingerprints of the structure and arrangement of a molecule. Hence it provides the position of the atom/molecule [86,87,88,89,90,91,92,93,94,95,96]. THz has been used to identify various chemical compounds, including amino acids and drug molecules. García-García et al., 2013 [90] performed Time Domain Tera Hertz spectroscopy (THz-TDS) of two drugs (paracetamol and ibuprofen), which resulted in different spectra [90]. The advantage of this low-energy radiation source is it does not ionize the objects, is easily controlled, and the frequency coincides with the frequency spectrum of important molecules [87,88,89,90,91,92,93,94,95,96]. Hence, THz is a potential tool for analyzing multicomponent development.

Last but not least, solid state nuclear magnetic resonance spectroscopy (SSNMR) has been used as an adequate analytical for pharmaceutical characterization, especially if a single crystal cannot be isolated [97,98,99,100,101,102,103]. This method can be used for qualitative and quantitative analysis [101,102,103,104,105,106,107,108]. But it is a sophisticated instrument; hence, only experts can operate and interpret the data. Nevertheless, it is applied in the pre-formulations, formulations, and manufacturing of pharmaceuticals [109,110,111,112,113,114]. This spectroscopy detected the interaction of the nuclei of atoms with radio electromagnetic (REM) waves by observing the resonance frequency yielded. The 500–1000 MHz frequency is used to measure the magnetogyric ratio constant, which is specific for each isotope. Hereafter, SSNMR can determine the structure, polymorphism, amorphous/crystalline, solid phase dynamic, and quantitation. Moreover, both methods can support the structure determination of polymorphism and multicomponent systems [114,115,116,117,118,119,120,121,122].

Afterward, the three dimensions structure can be precisely predicted using SCXRD by mapping each electron and calculating their position, conformation, and bond angle. SCXRD required a regular and pure phase of the sample, named single crystal, which consists of similar molecules in a uniform order of the symmetrical pattern arrangement with a homogenous lattice structure [59]. The solvent evaporation method can produce the appropriate crystals optimally, which are then selected by observation under a microscope. SCXRD provides data about the lattice’s length and angle, crystal packing in point of view a, b, and c, entirely [10,123,124,125,126,127].

Besides identifying the new solid phase qualitatively, recently, kinetic and stability studies of the cocrystal formation of a drug were successfully done using diffraction instruments and thermal analysis [128,129,130,131]. The area under the curve of a thermogram represented the proportion of a substance in a sample. Therefore, both methods are utilized for measuring the complementary concentration.

## 4. Antiviral Multicomponent System

In these past ten years, nevirapine and curcumin have been reported as the most developed antiviral to multicomponent systems such as cocrystals. In the nevirapine structure, an N-H group near the ketone group has a primary role in the multicomponent formation. This group is the site where nevirapine and its coformers were bonded. Most coformers used were carboxylic group compounds, and the weak hydrogen bond formed the multicomponent compounds, the interaction displayed in Table 2 (No. 39–44). It was detected by FTIR spectroscopy that showed bands shifting that indicated C=O, O-H, and N-H groups in the nevirapine structure and C=O group in the coformer’s network [83]. Since nevirapine is classified into BCS class 2, it has low solubility in water, and its multicomponent form increased its solubility. The coformer’s properties caused it, most of the coformers are weak acids that easily dissociate. The solubility enhancement of nevirapine led to bioavailability and dissolution rate increase [83].

Meanwhile, in the curcumin structure, two ketone groups are likely to form weak hydrogen bonds with the hydroxy groups in the coformers. Curcumin is commonly combined with the amino acids coformer since hydroxy groups are in the structure. Curcumin is one of the most developed antivirals to be a multicomponent compound, caused by it is provided two sites of hydrogen bond formation [132]. The interaction between curcumin and amino acids is displayed in Table 2 (No. 10–17). Curcumin multicomponent compounds also increase the solubility of curcumin since the amino acid coformers are likely to form H-bonding through primary amine and charged groups. The solubility enhancement of curcumin leads to a low dose of curcumin in a pharmaceutical dosage form, which can reach therapeutic plasma concentrations after oral administration [132].

**Figure 5:**
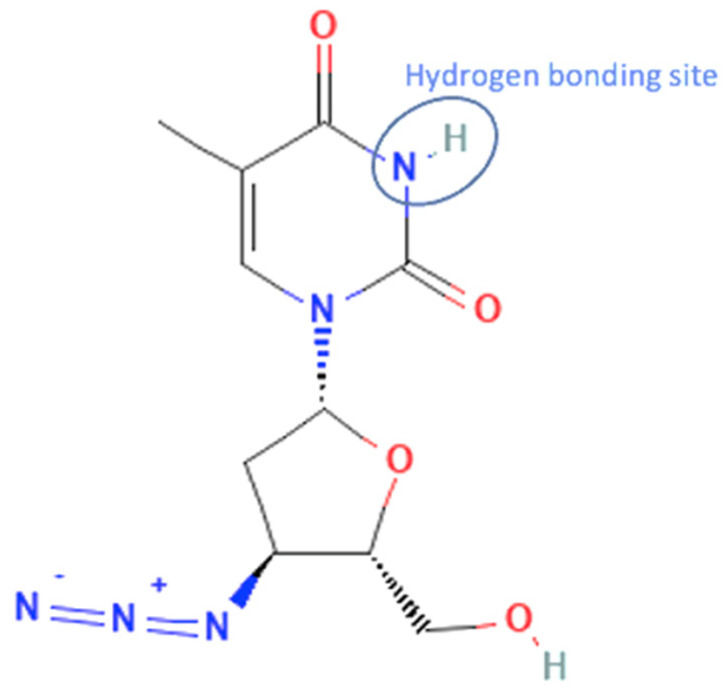
Molecular structure of zidovudine and its interaction site [133].

On the other hand, there are also the least antivirals that developed to be multicomponent compounds. First, zidovudine has a nitrogen group in its structure. That is where zidovudine and its coformer interact to form hydrogen bonds, as in the zidovudine—picric acid system. Zidovudine was the least antiviral that succeeded in forming a multicomponent system caused by it only has one nitrogen group/bonding site of the hydrogen bonds. The zidovudine multicomponent compound increased the temperature stability, reducing storage temperature and half-life [56]. However, the lamivudine-zidovudine multicomponent system has become a famous drug for HIV treatment [20]. The molecular structure and bonding site of zidovudine can be seen in Figure 5.

Second, lamivudine was classified as the least developed antiviral because it has several unstable polymorphs with various properties. However, each lamivudine polymorph has a different interaction and bond formation ability. The successful multicomponent lamivudine was the lamivudine polymorph II combined with theophylline as a coformer. The multicomponent compound was formed by the interaction between the amine group of lamivudine and the carbonyl group of theophylline, forming a hydrogen bond [56]. The interaction between lamivudine and theophylline can be seen in Table 2 (No. 59). The advantage of lamivudine multicomponent formation was the stability maintenance of lamivudine, which leads to better safety and efficacy [64]. Many researchers have developed antiviral multicomponents, and their advantages are summarized in Table 2.

**Table 2:** Antiviral Multicomponent Systems.

No.	Advantage categories	Multicomponent	Structure	Advantages	Preparation methods	Ref.
1	Increasing solubility and dissolution rate	Arbidol-maleic acid	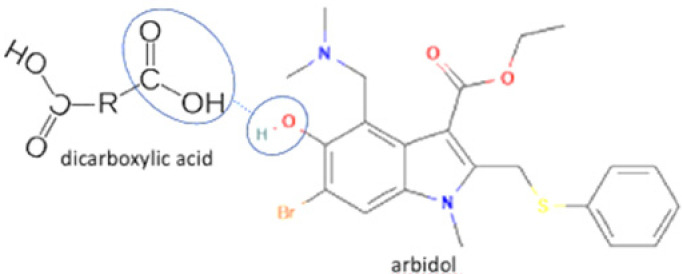	Increasing the solubility and dissolution of arbidol alone	Slow evaporation	[72]
2		Arbidol-fumaric acid		Increasing the solubility and dissolution of arbidol alone	Slow evaporation	[72]
3		Favipiravir-piperazine	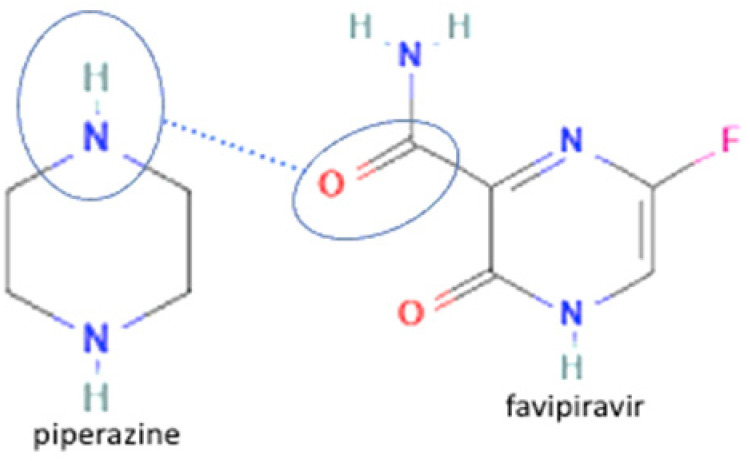	Enhanced the solubility of favipiravir in pH 6.8 by 1.6-fold and enhanced the tabletability of favipiravir	Slow evaporation	[131]
4		Favipiravir-4 dihydroxy benzoic acid	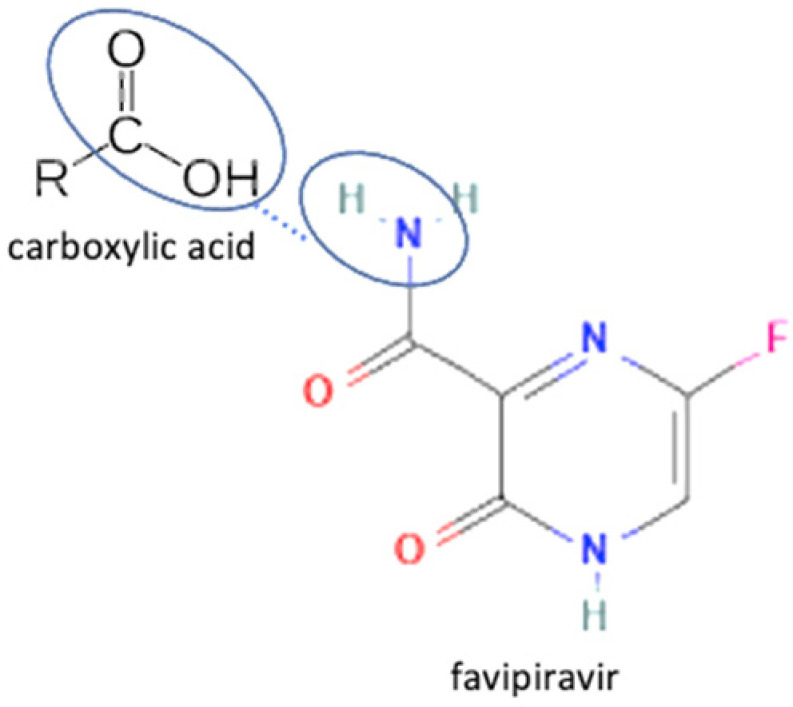	Solubility improvement of favipiravir in distilled water	Liquid assisted grinding	[134]
5		Favipiravir-gallic acid		Solubility improvement of favipiravir in buffer phosphate pH 7	Liquid assisted grinding	[134]
6		Favipiravir-4 amino benzoic acid		Solubility improvement of favipiravir in distilled water and in buffer phosphate pH 7	Liquid assisted grinding	[134]
7		Curcumin-ascorbic acid	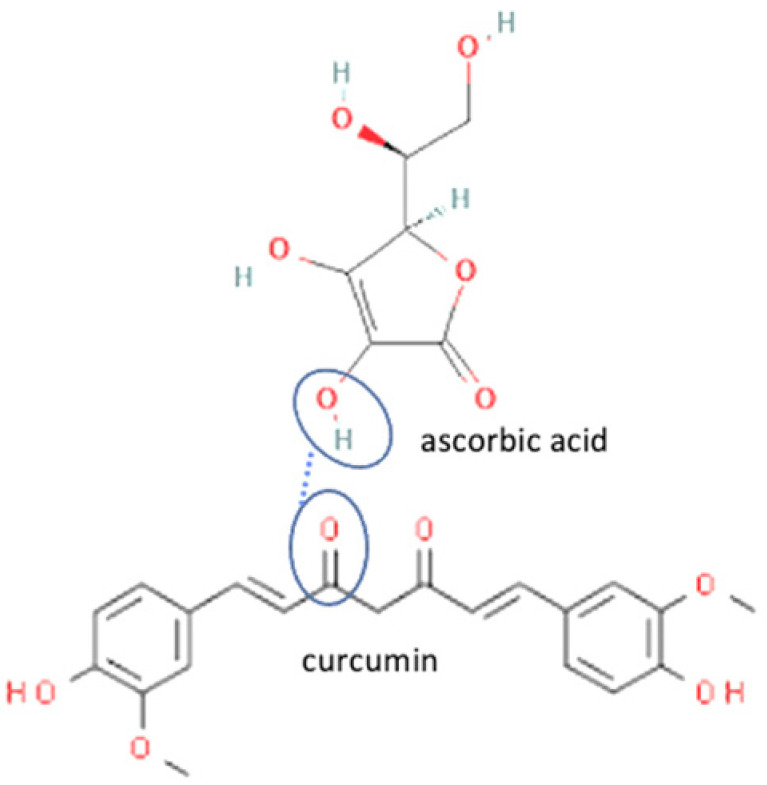	Enhanced the aqueous solubility of curcumin in distilled water, pH 1.2, and pH 6.8 by 576, 10, and 9 fold, respectively. Enhanced the dissolution profile of neat curcumin	Solvent evaporation	[132]
8		Abacavir-oxalic acid	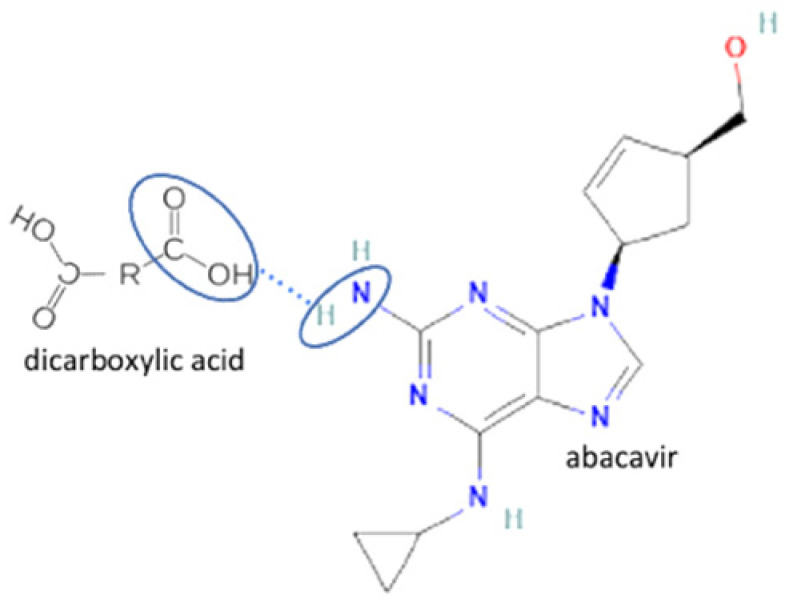	Enhancing the aqueous solubility and dissolution rate of abacavir	Solvent evaporation	[135]
9		Abacavir-glutaric acid		Enhancing the aqueous solubility and dissolution rate of abacavir	Solvent evaporation	[135]
10		Curcumin-tyramine	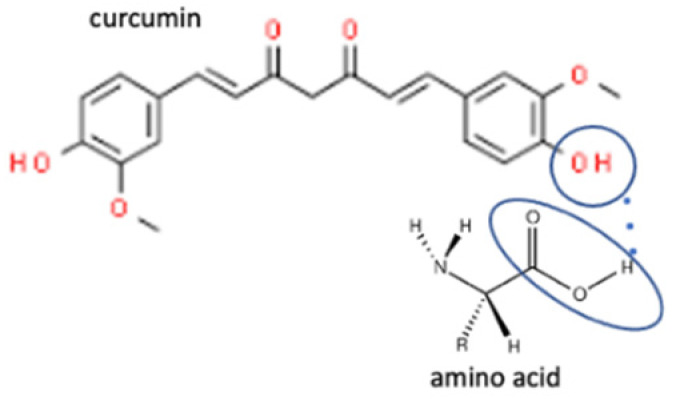	Increasing the solubility by 18.6 times in a 40% ethanol medium of curcumin	Liquid assisted grinding	[132]
11		Curcumin-tryptamine		Increasing the solubility by 6.3 times in a 40% ethanol medium of curcumin	Liquid assisted grinding	[132]
12		Curcumin-arginine		Increasing the solubility by 9.1 times in a 40% ethanol medium of curcumin	Neat grinding	[132]
13		Curcumin-asparagine		Increasing the solubility by 1 time in a 40% ethanol medium of curcumin	Neat grinding	[132]
14		Curcumin-glutamine		Increasing the solubility by 1.1 times in a 40% ethanol medium of curcumin	Neat grinding	[132]
15		Curcumin-lysine		Increasing the solubility by 1.1 times in 40% ethanol medium of curcumin	Neat grinding	[132]
16		Curcumin-histidine		Increasing the solubility by 4.2 times in 40% ethanol medium of curcumin	Neat grinding	[132]
17		Curcumin-citrulline		Increasing the solubility by 1.1 times in 40% ethanol medium of curcumin	Neat grinding	[132]
18		Quercetin-succinic acid	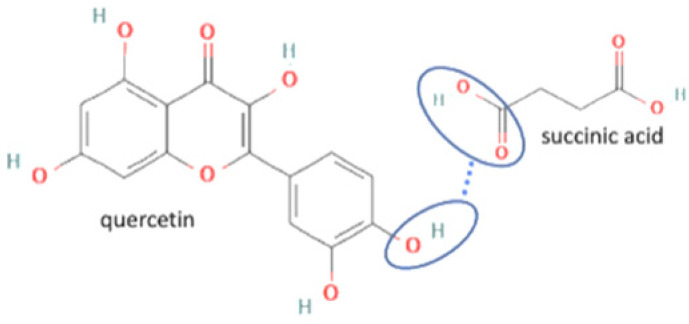	Increasing the solubility and dissolution rate by 16.2 and 1.25 times, respectively of quercetin alone	Liquid assisted grinding	[136]
19		Quercetin-isonicotinamide	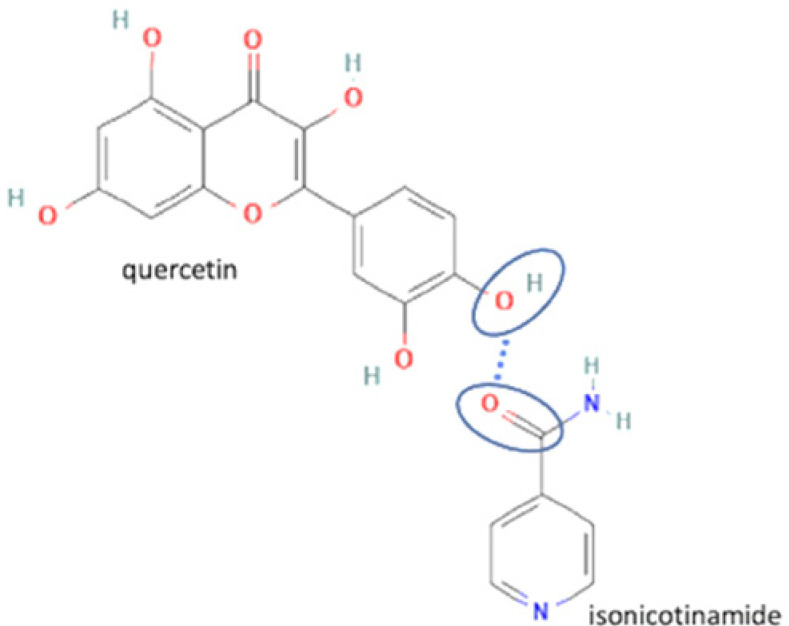	Dissolution profile improvement of quercetin alone	Solvent evaporation	[137]
20		Sulfathiazole-amantadine	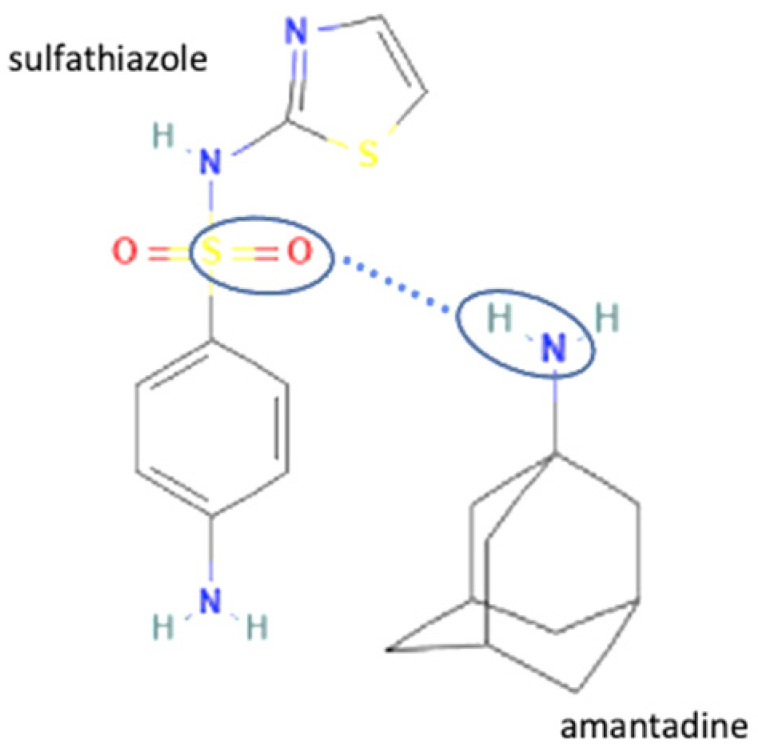	Solubility improvement, diffusion improvement, dissolution improvement (2x), antibacterial activity improvement,	Liquid-assisted grinding followed by solvent evaporation	[51]
21		Arbidol-succinic acid	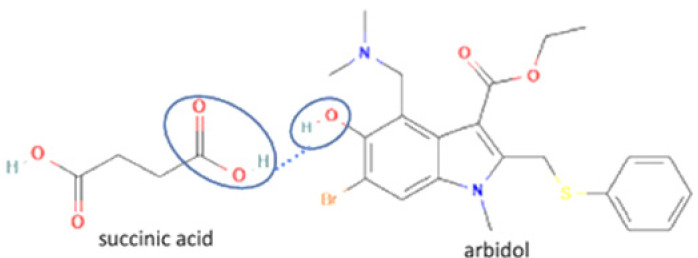	Solubility improvement (7x in pH1.2), dissolution profile improvement	Liquid-assisted grinding followed by solvent evaporation	[72]
22		Arbidol-salicylic acid	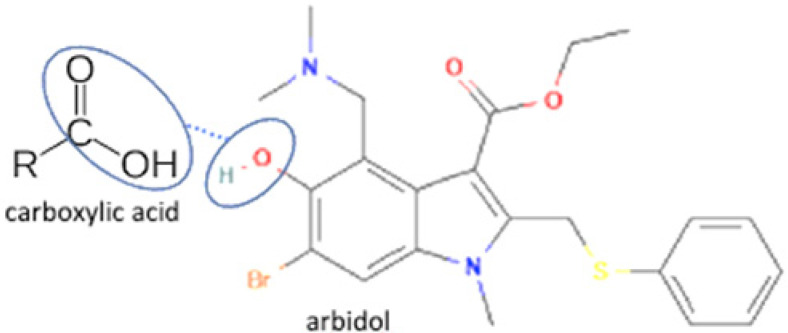	Solubility improvement (3x), lower energy	Slow evaporation	[40]
23		Arbidol-benzoic acid		Solubility improvement (3x), lower energy	Slow evaporation	[40]
24		Acyclovir-gallic acid	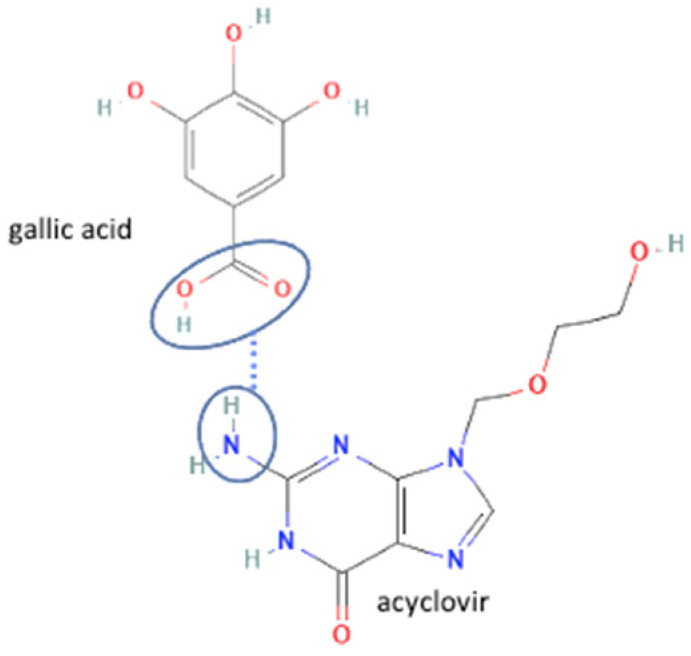	Solubility and dissolution rate improvement	Cogrinding	[138]
25		Efavirenz-oxalic acid	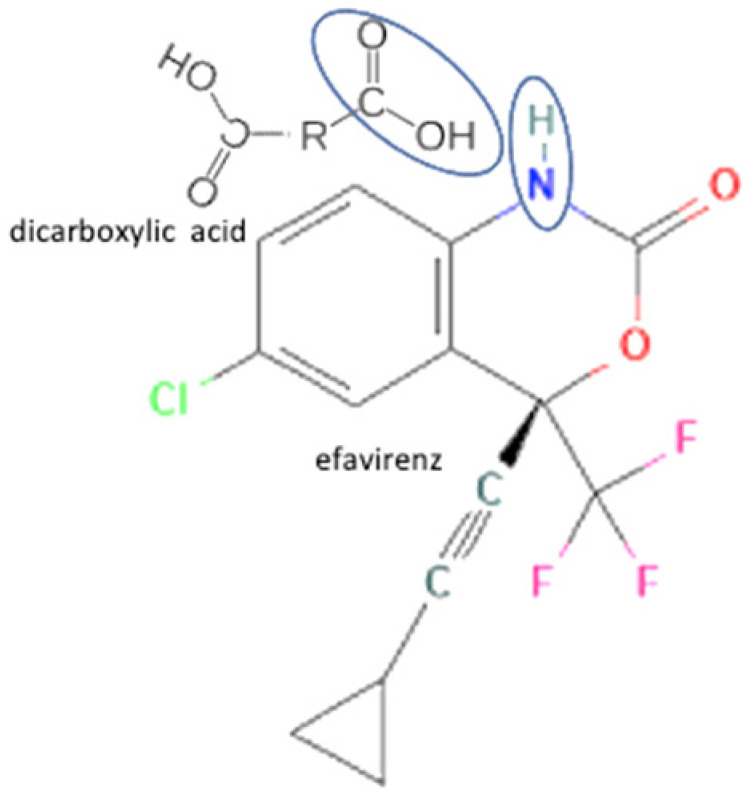	Lower melting point, new hydrogen bond addition, solubility improvement	Cogrinding	[139]
26		Efavirenz-glutaric acid		Solubility improvement, improvement of drug release	Spray and freeze drying	[62]
27		Efavirenz-citric acid	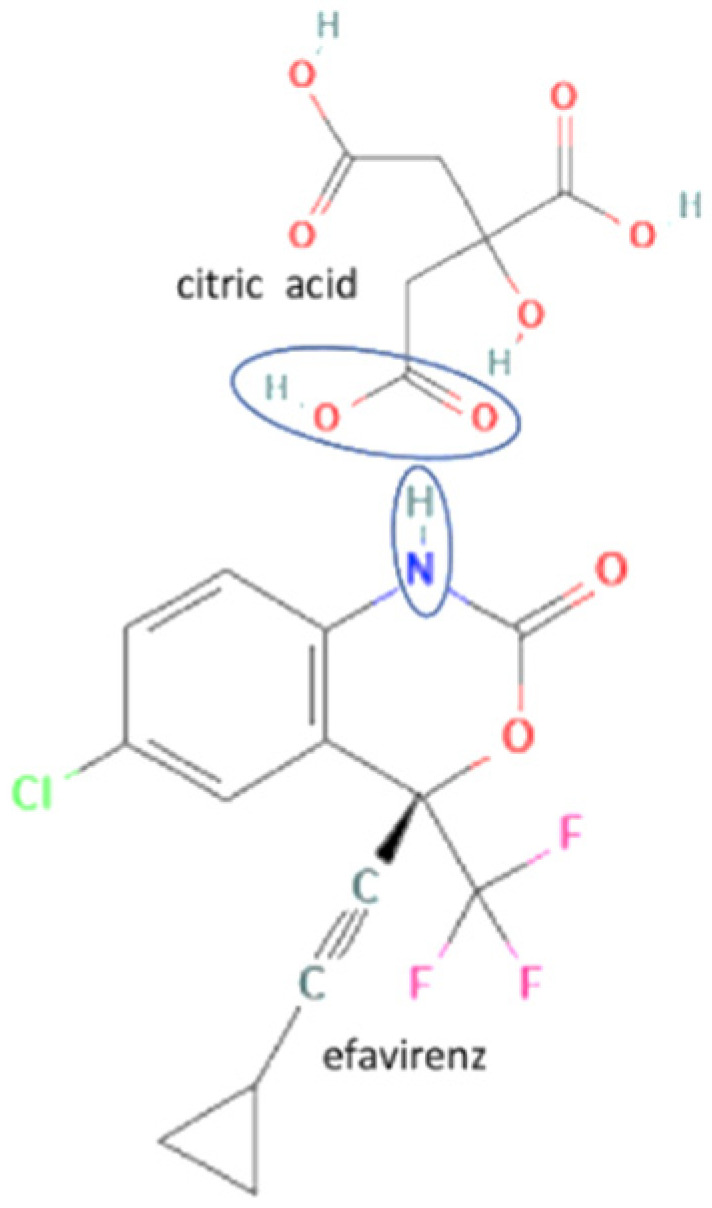	Lower melting point, new hydrogen bond addition, solubility improvement	Cogrinding	[139]
28		Acyclovir-tartaric acid	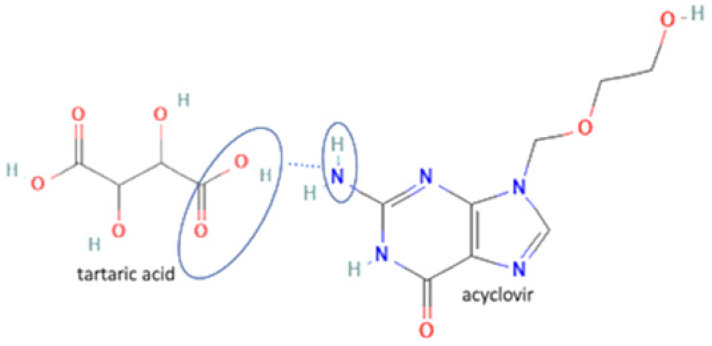	Solubility improvement	Solution evaporation and grinding technique	[140]
29		Etravirine-adipic acid	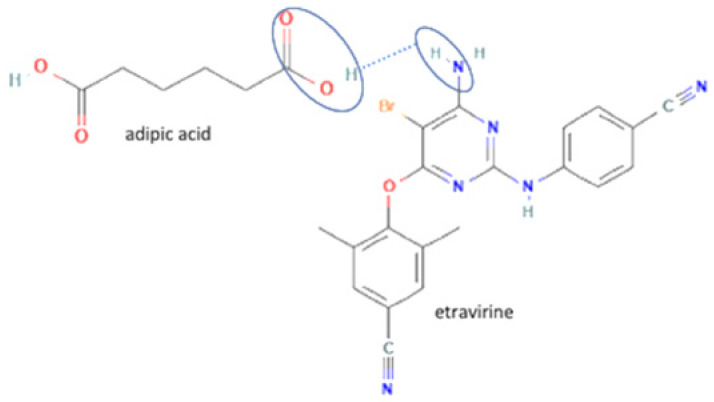	Solubility improvement, improvement of drug release	Solvent evaporation	[141]
30		Penciclovir-3.5 dihydroxy benzoic acid	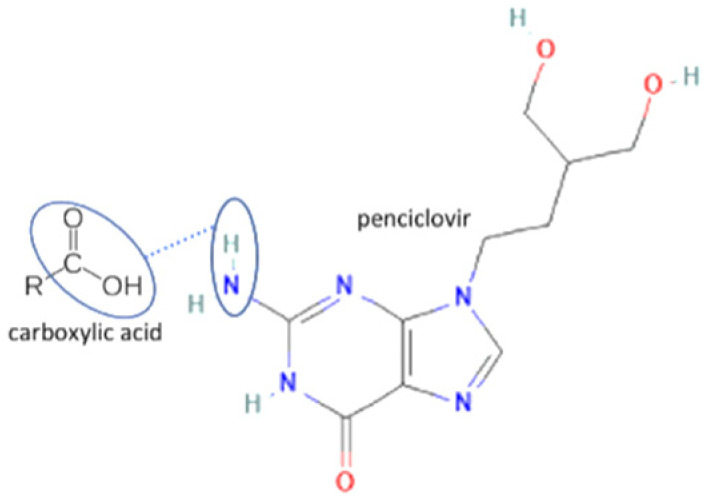	Solubility improvement, hydrogen bond addition, maintaining stability	Neat grinding	[55]
31		Penciclovir-gallic acid		Solubility improvement, hydrogen bond addition, maintaining stability	Neat grinding	[55]
32		Penciclovir-4 hydroxycinnamic acid		Solubility improvement, hydrogen bond addition, maintaining stability	Neat grinding	[55]
33		Ritonavir-nicotinamide	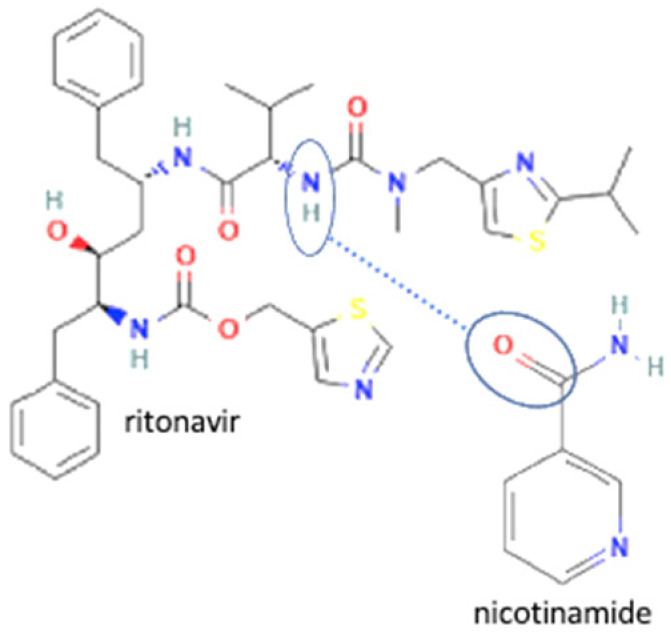	Solubility improvement (3–4 x)	Liquid assisted grinding	[142]
34		Ritonavir-succinic acid	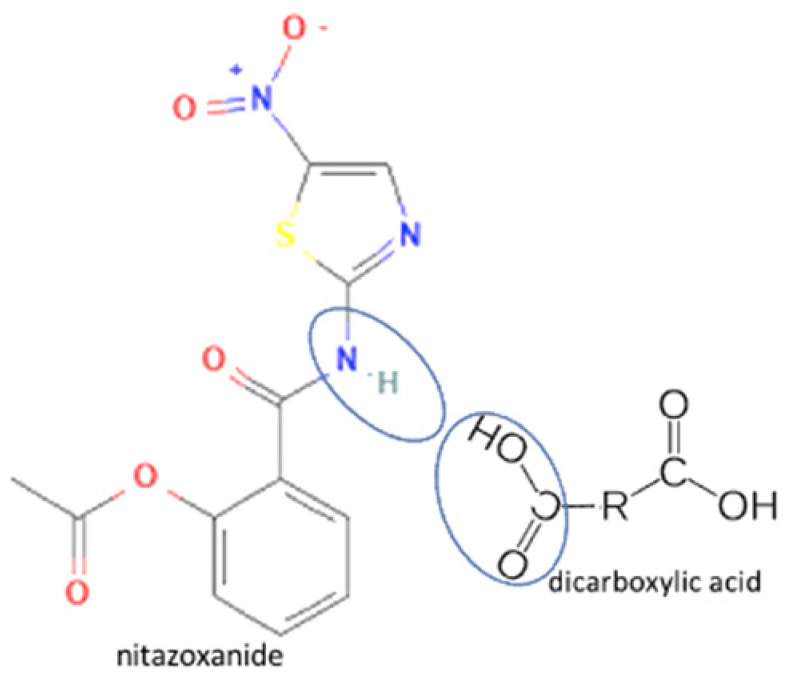	Solubility improvement (3–4 x)	Liquid assisted grinding	[142]
35		Ritonavir-adipic acid		Solubility improvement (3–4 x)	Liquid assisted grinding	[142]
36		Ritonavir-d-alanine	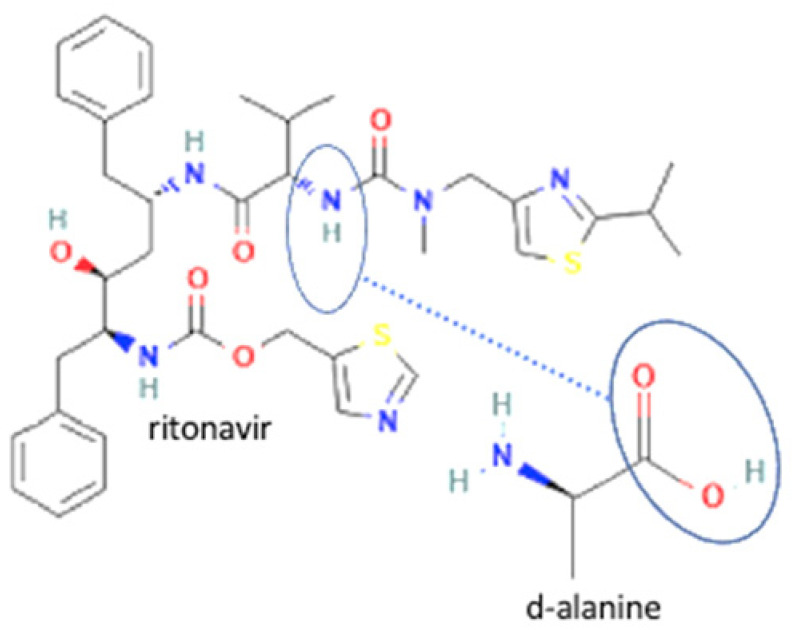	Solubility improvement (3–4 x)	Liquid assisted grinding	[142]
37		Nevirapine-saccharin	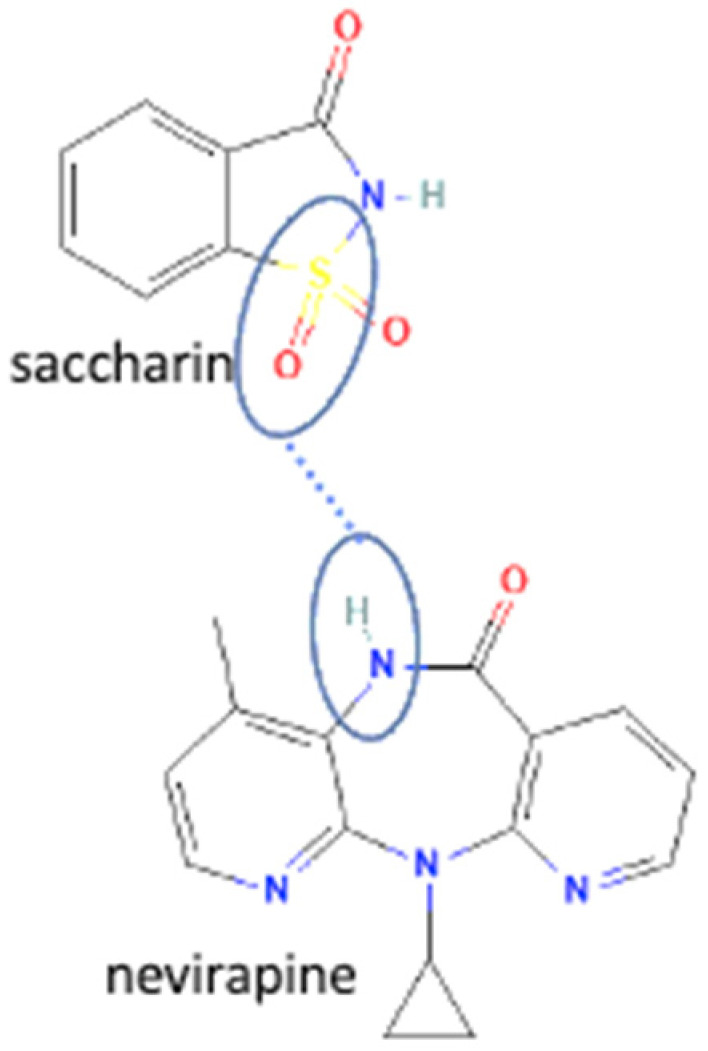	Hydrogen bond formation [95], dissolution rate enhancement	kneading, solution crystallization, antisolvent addition, and solvent drop grinding	[83]
39		Nevirapine-tartaric acid	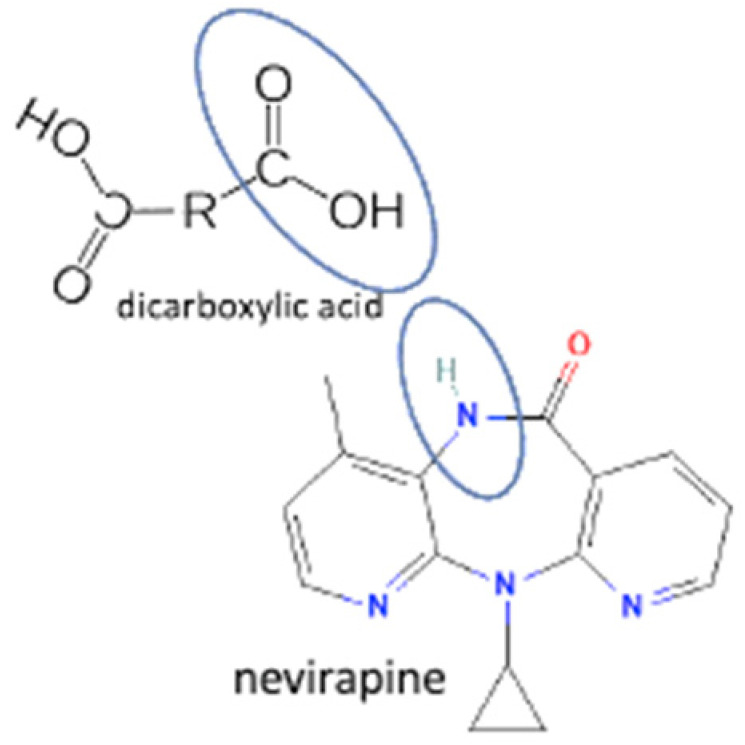	Hydrogen bond formation, dissolution rate enhancement	liquid-assisted grinding	[83]
40		Nevirapine-maleic acid		Hydrogen bond formation, dissolution rate enhancement	liquid-assisted grinding	[83]
41		Nevirapine -glutaric acid		Hydrogen bond formation, dissolution rate enhancement	liquid-assisted grinding	[83]
42		Nevirapine-salicylic acid		Hydrogen bond formation, dissolution rate enhancement	kneading, solution crystallization, antisolvent addition, and solvent drop grinding	[83]
43		Nevirapine -3 hydroxybenzoic acid	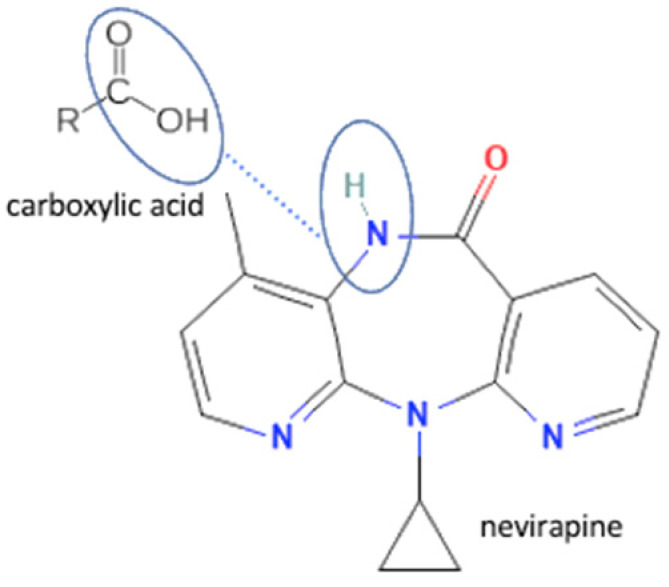	Hydrogen bond formation, dissolution rate enhancement	liquid-assisted grinding	[83]
44		Nevirapine -4 hydroxybenzoic acid		Hydrogen bond formation, dissolution rate enhancement	liquid-assisted grinding (LAG)	[83]
45		Nevirapine -theophylline	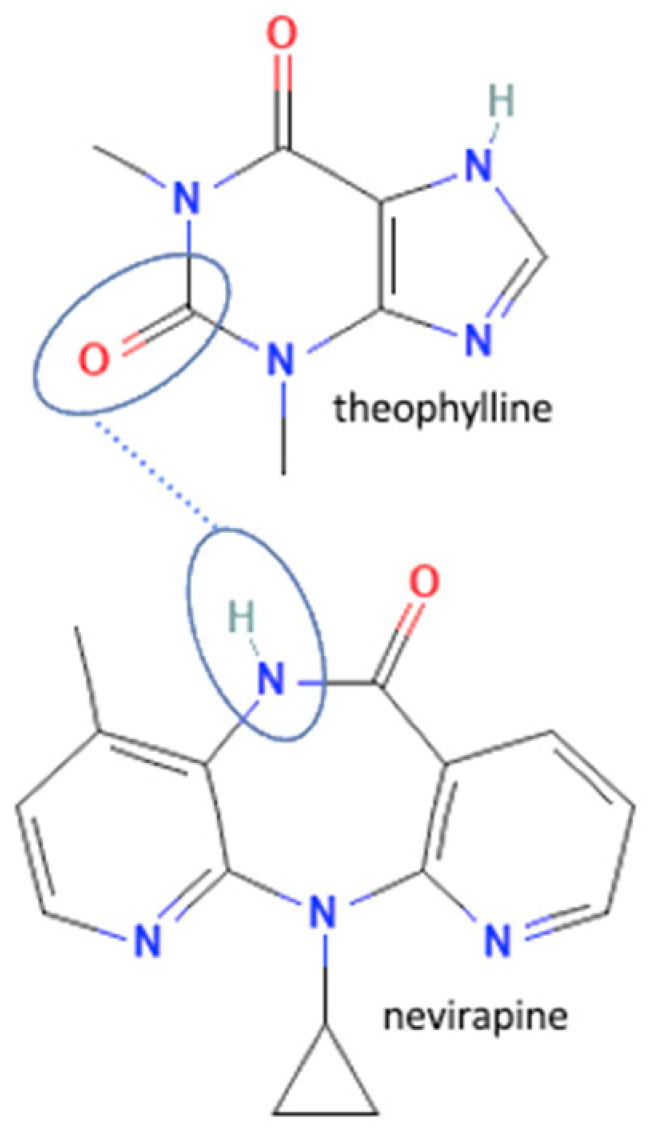	Hydrogen bond formation, dissolution rate enhancement	liquid-assisted grinding	[83]
46		Nevirapine -caffeine	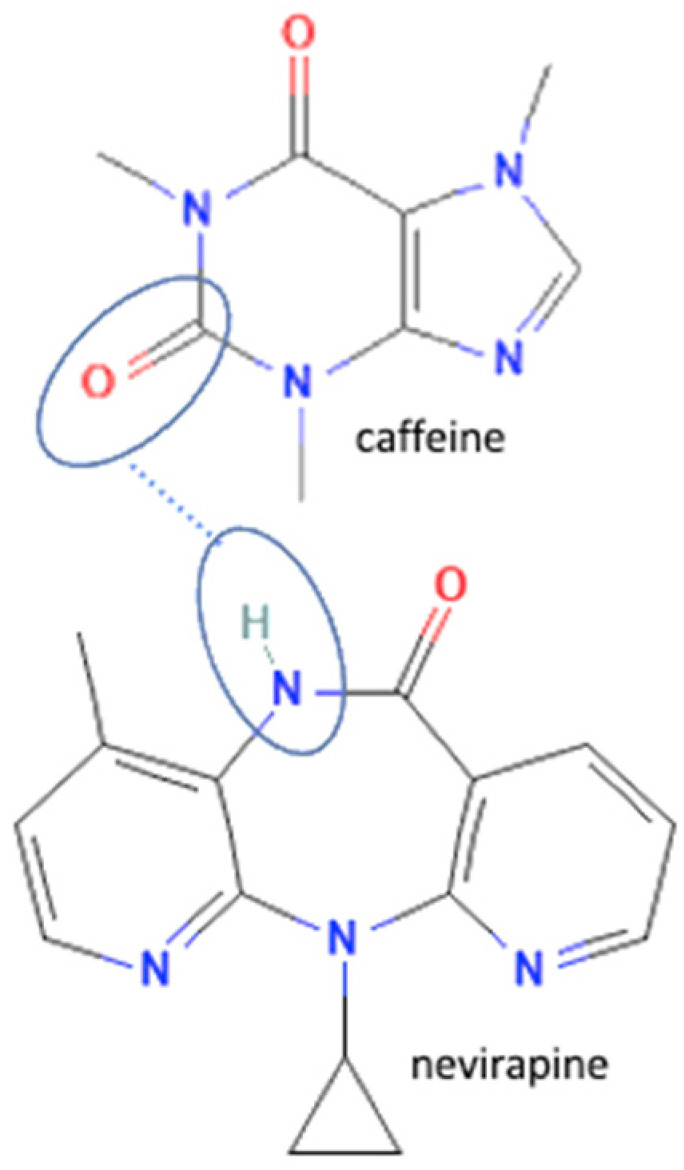	Hydrogen bond formation, dissolution rate enhancement	liquid-assisted grinding	[83]
47		Nitazoxanide-glutaric acid	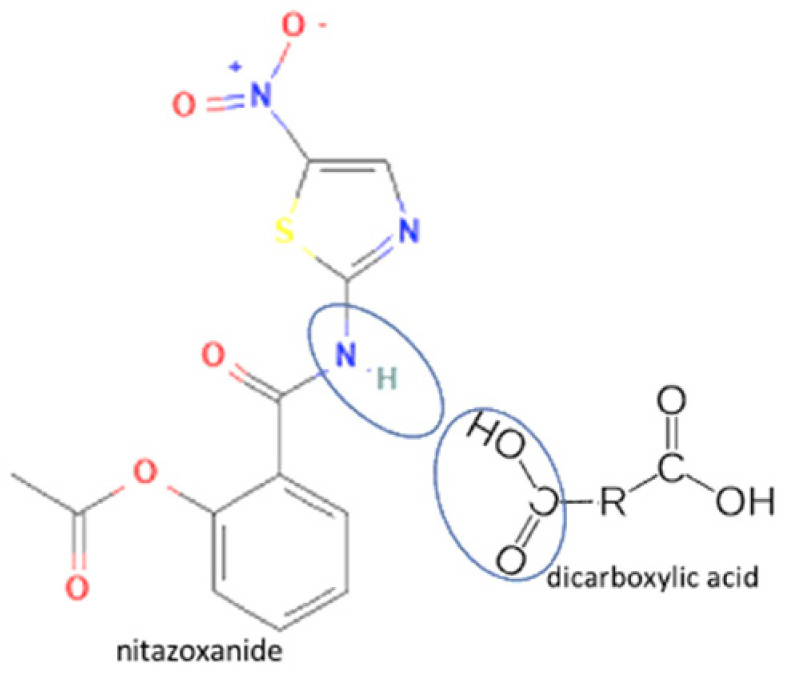	Dissolution properties enhancement [97]	Neat grinding	[77]
48		Nitazoxanide -succinic acid		Dissolution properties enhancement [97]	Neat grinding	[77]
49	Increasing stability	Efavirenz-tartaric acid	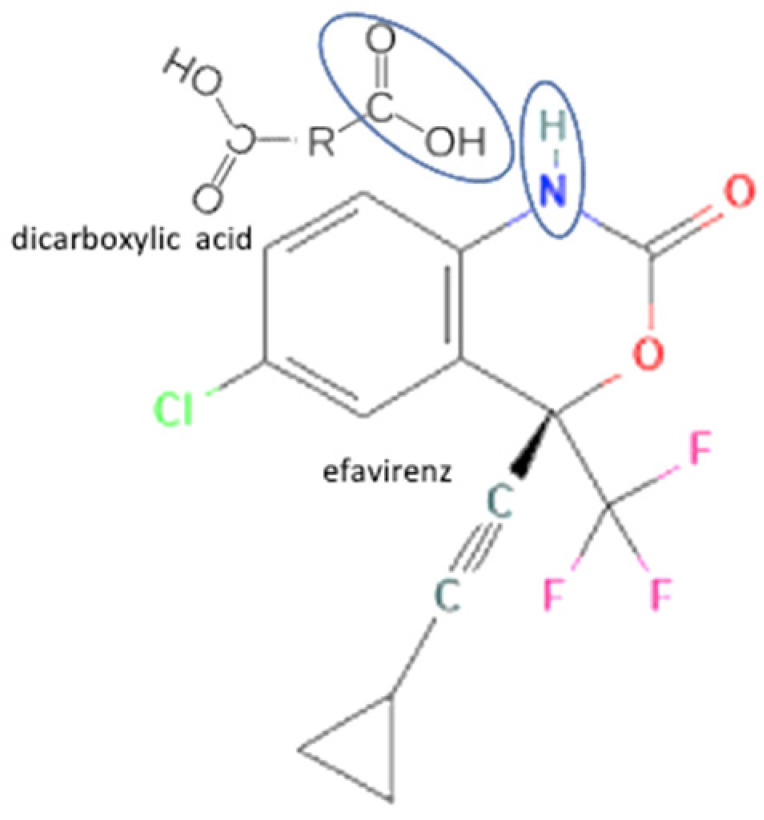	Enhanced the solubility by 1.8-fold and dissolution rate by 1.4 times of efavirenz alone, also increasing stability		[143]
50		Efavirenz-adipic acid		Enhanced the solubility by 1.2-fold and dissolution rate by 1.2 times of efavirenz alone, also increasing stability	Slow evaporation	[143]
51		Penciclovir-3.5 hydroxybenzoic acid	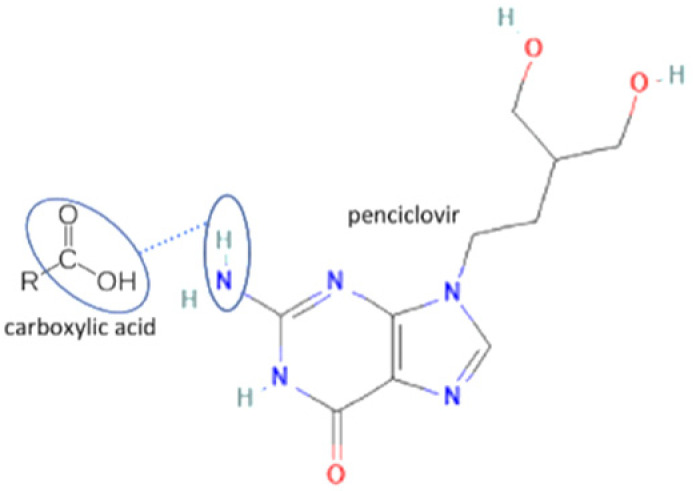	Increasing the solubility by 129% and increasing the stability of penciclovir	Slurry method	[55]
52		Penciclovir-gallic acid		Increasing the solubility by 29% and increasing the stability of penciclovir	Liquid-assisted grinding and slurry	[55]
53		Dipyridamole-tartaric acid	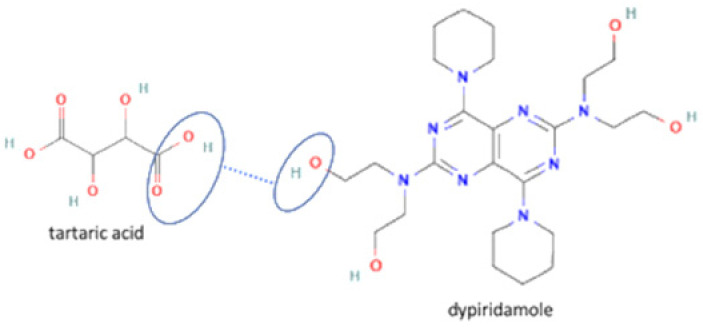	Solubility and stability improvement of dipyridamole alone	Liquid assisted grinding	[144]
54		Adefovir-gallic acid	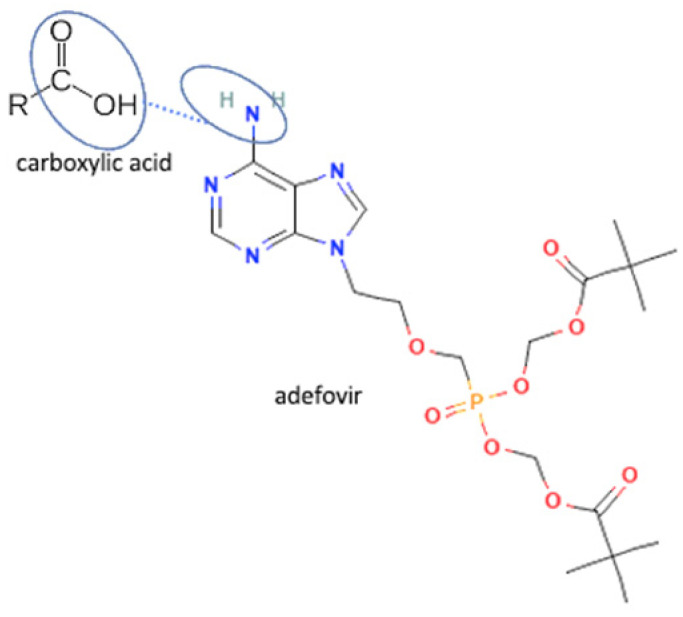	Stability improvement	Liquid assisted grinding	[37]
55		Adefovir-salicylate		Stability improvement	Slow evaporation	[37]
56		Adefovir-saccharin	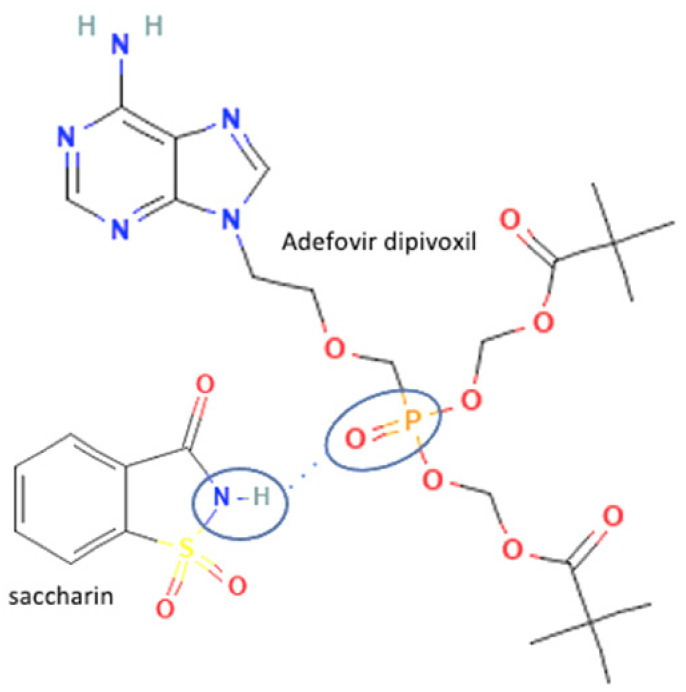	Shelf-life enhancement of adevofir	Slow evaporation	[37]
57		Acyclovir-fumaric acid	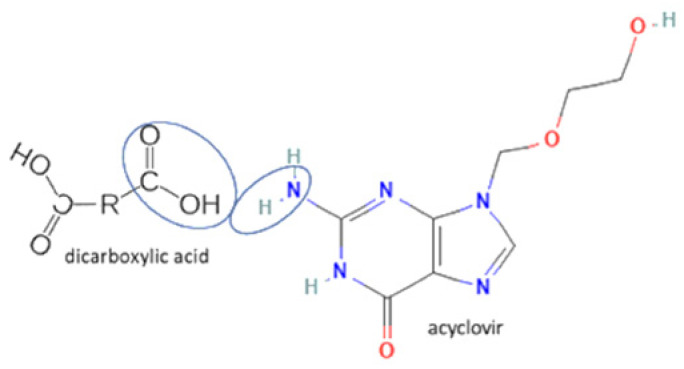	Cocrystal stability improvement, solubility improvement, dissolution profile improvement.	Solution evaporation and grinding technique	[140]
58		Acyclovir-maleic acid		Cocrystal stability improvement, solubility improvement acyclovir release from the higher crystal	Solution evaporation and grinding technique	[140]
59		Lamivudine-theophylline (polymorph 1)	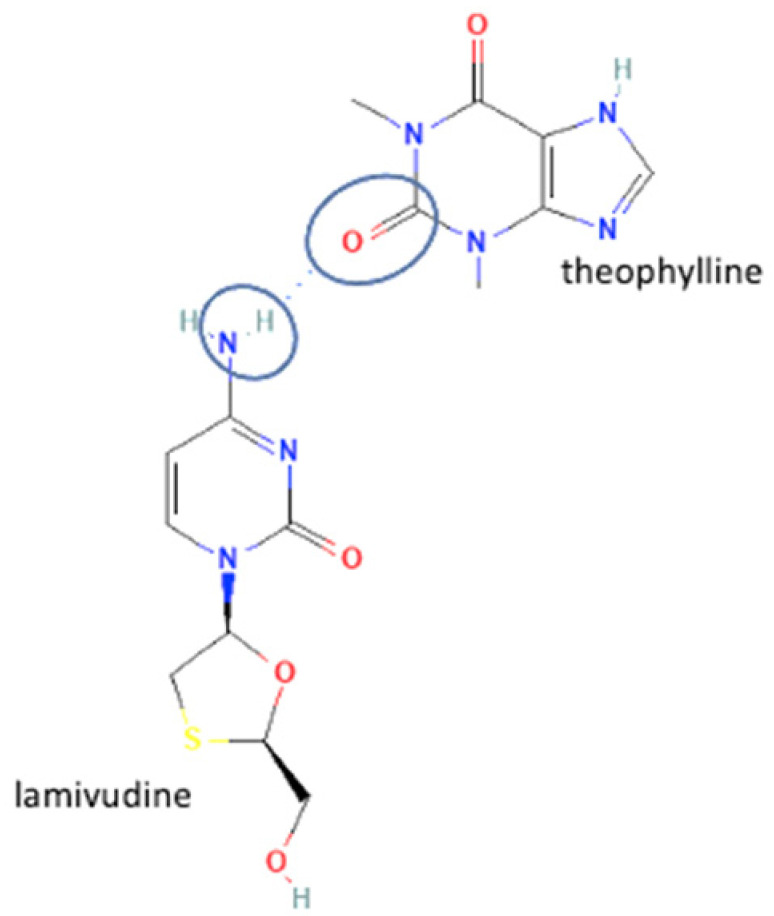	Maintaining the stability of lamivudine	Neat grinding and liquid-assisted grinding	[62]
60		Zidovudine-picric acid	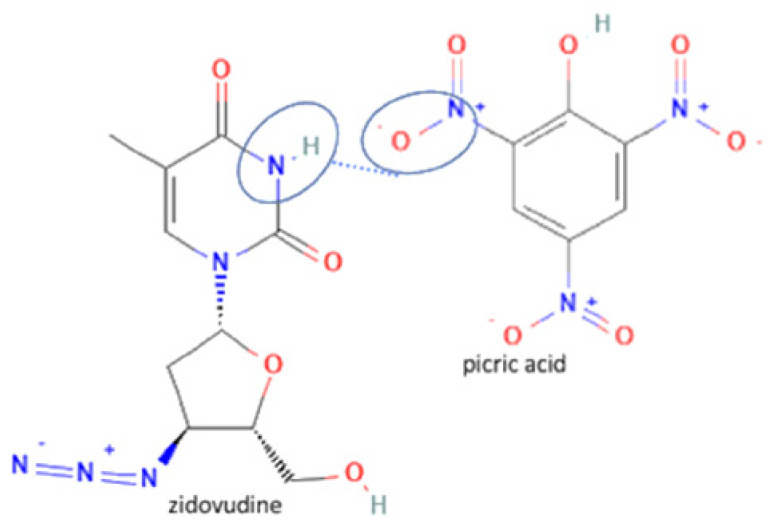	Stability improvement at 129 °C	Slow evaporation	[62]
61	Increasing stability toward moisture	Favipiravir-theophylline	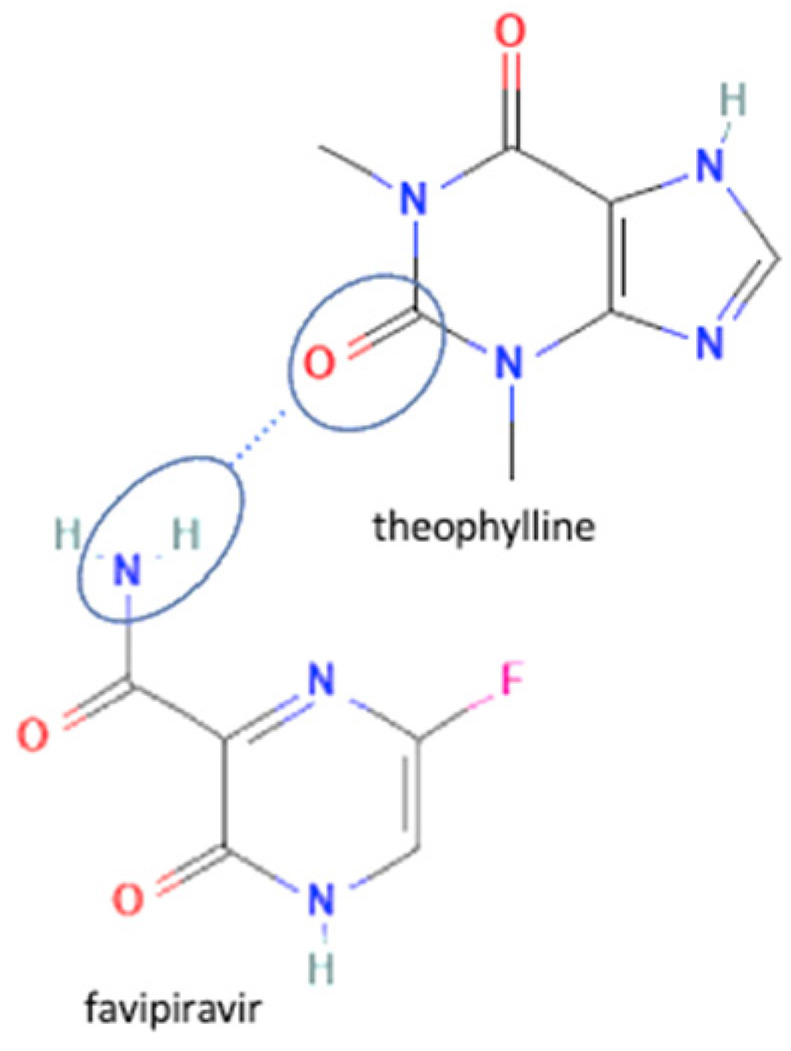	Solubility improvement of favipiravir in distilled water and buffer phosphate pH 7, stability improvement towards moisture of favipiravir	Liquid assisted grinding	[131]
62	Increasing bioavailability	Amantadine hydrochloride-resveratrol	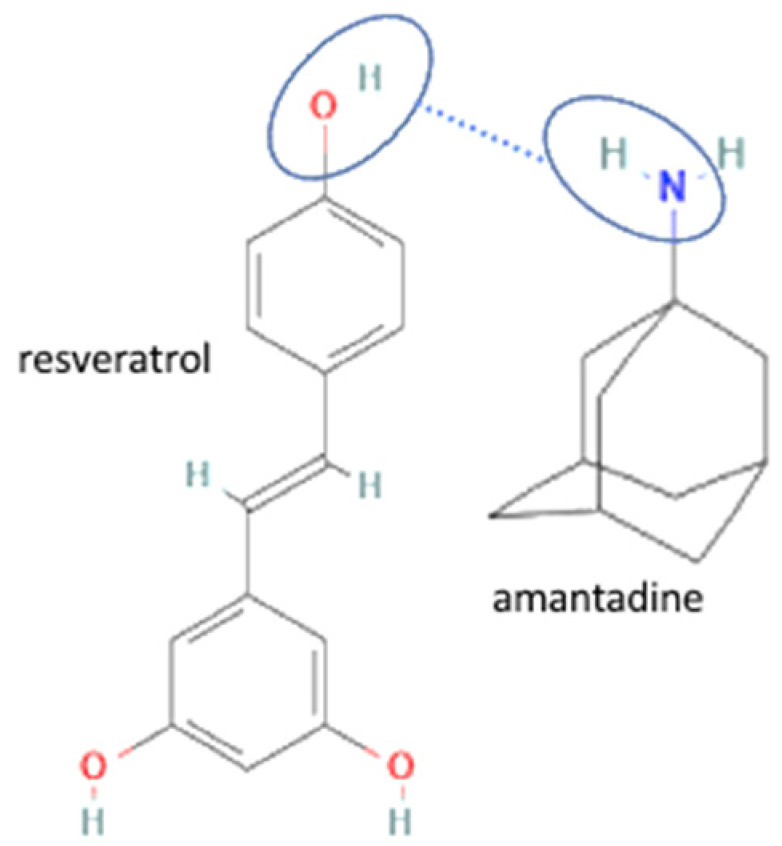	Increasing solubility and bioavailability 152 and 9.64 times compared to resveratrol alone, also achieving a synergistic antiviral efficacy.	Liquid-assisted grinding and solvent ultrasonic	[145]
63		Ribavirin-3.5 dihydroxy benzoic acid	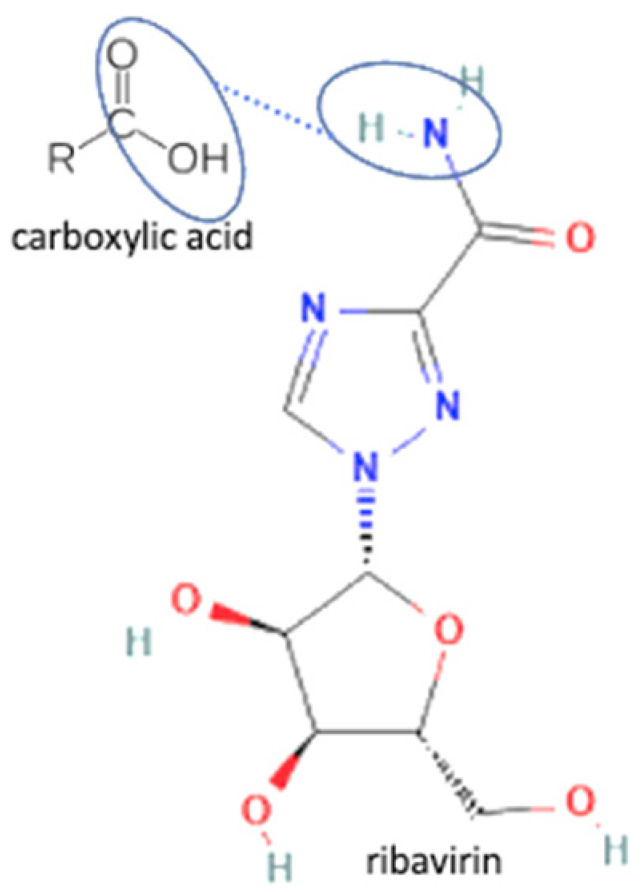	Enhance drug release of riboflavin	Slurry method	[146]
64		Ribavirin-gallic acid		Enhance drug release of riboflavin	Slurry method	[146]
65		Ribavirin-barbituric acid	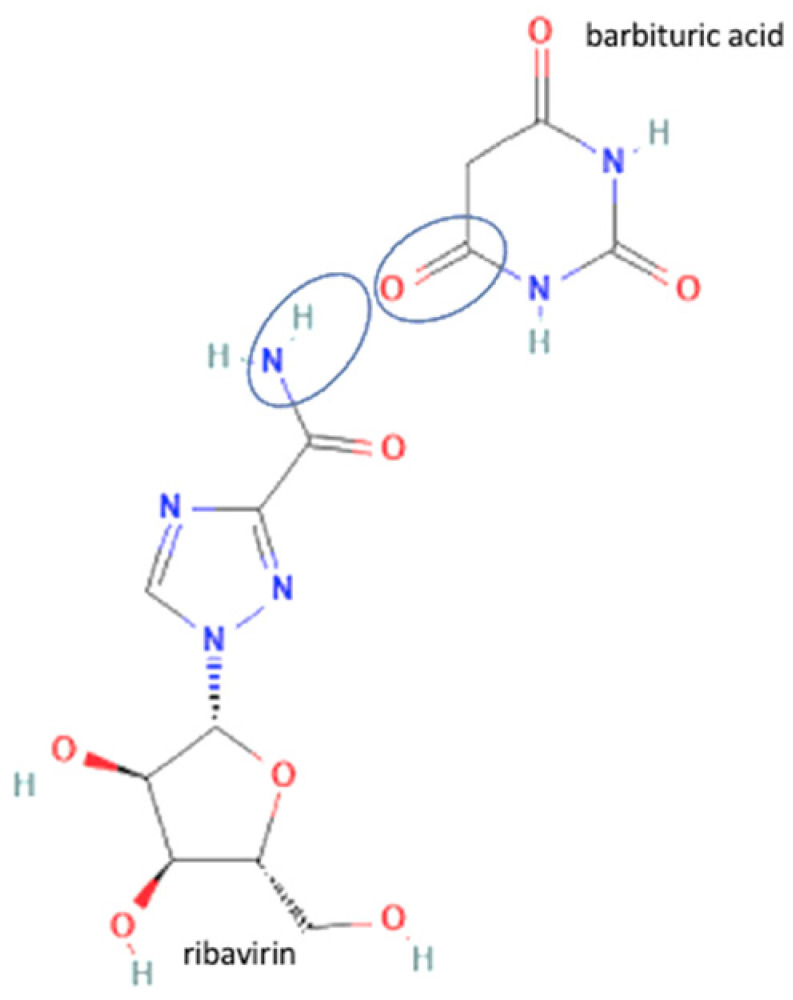	Enhance drug release of riboflavin	Slurry method	[146]
66		Emodin-nicotinamide acid	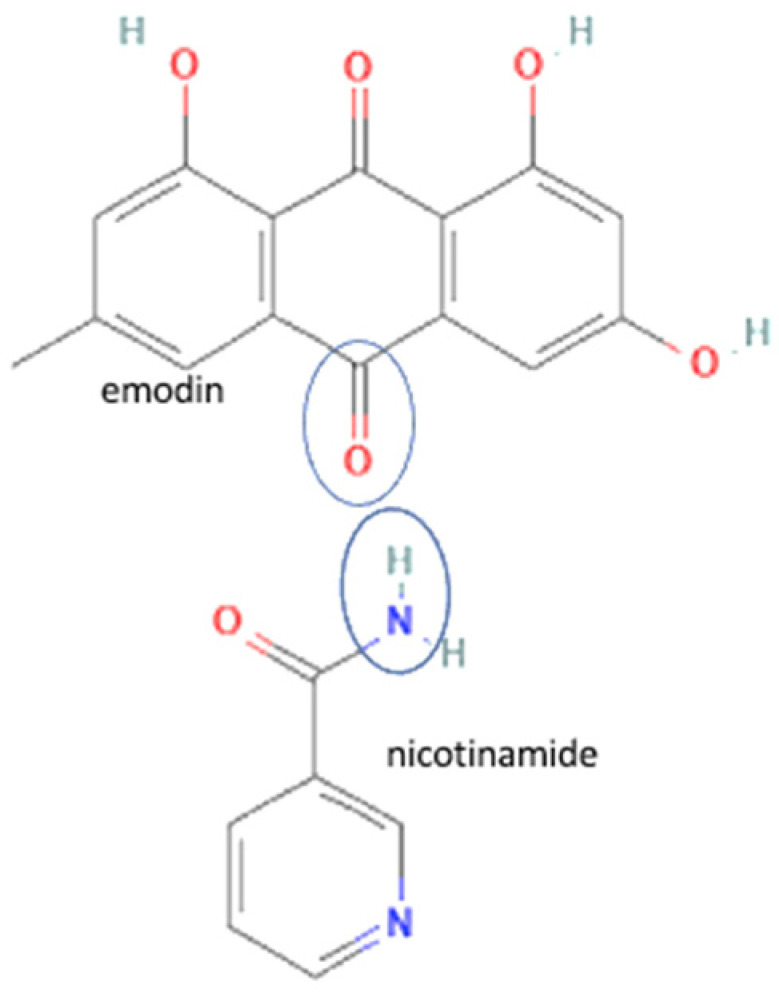	Drug release enhancement, stability towards humidity, and high-temperature stability improvement of emodin	Hot melt extrusion	[70]
67	Increasing Penetrability	Sulfathiazole-amantadine hydrochloride	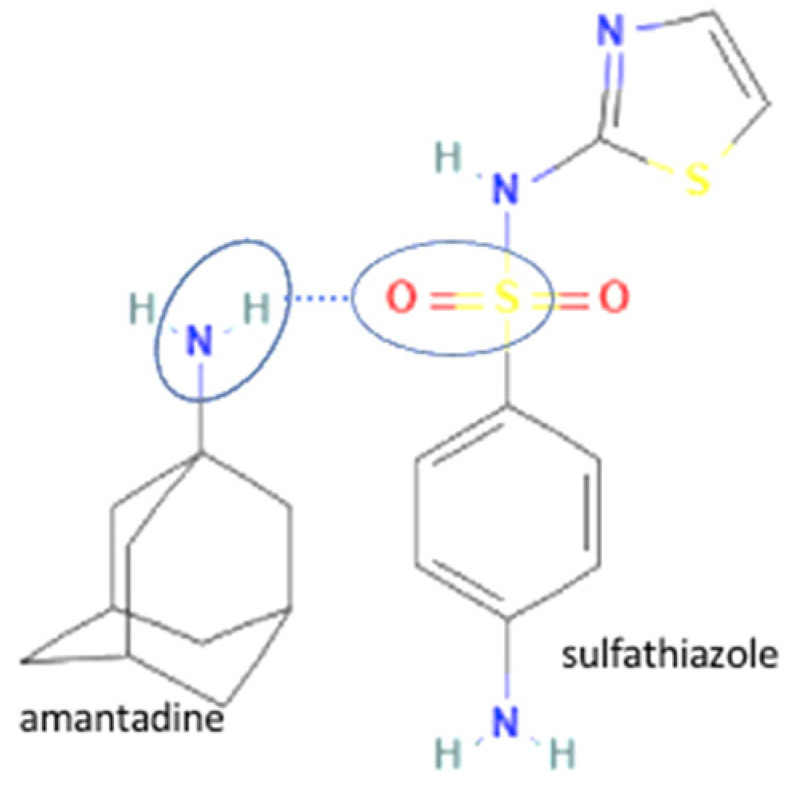	Water solubility improvement 1.83–5.23 times and 2-fold enhancement in penetrability of sulfathiazole alone	Liquid-assisted grinding and solvent evaporation	[147]
68	Increasing antiviral efficacy	Amantadine hydrochloride-ferulic acid	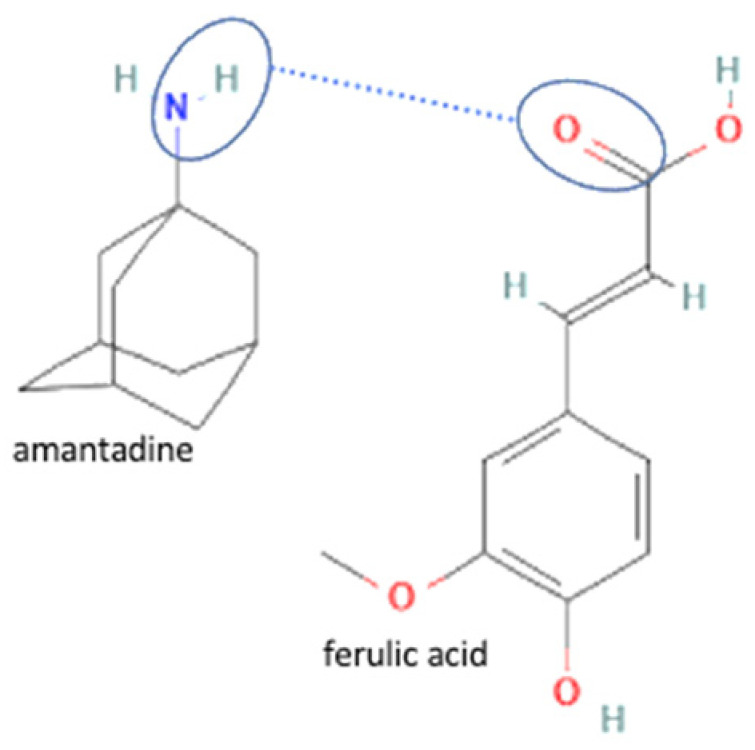	Dissolution improvement of amantadine hydrochloride 2–3 folds and enhanced the antiviral effects with a combination index <1	Liquid-assisted grinding and solvent evaporation	[148]
69	Increasing permeability	Favipiravir-saccharin	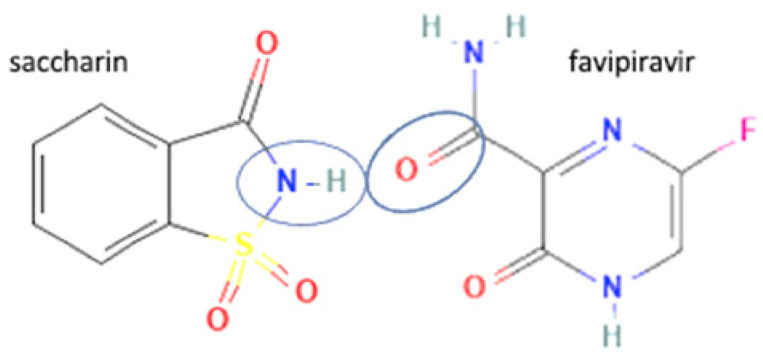	Enhanced the permeability and tablet ability of favipiravir	Liquid assisted grinding	[134]
70		Favipiravir-5 fluorouracil	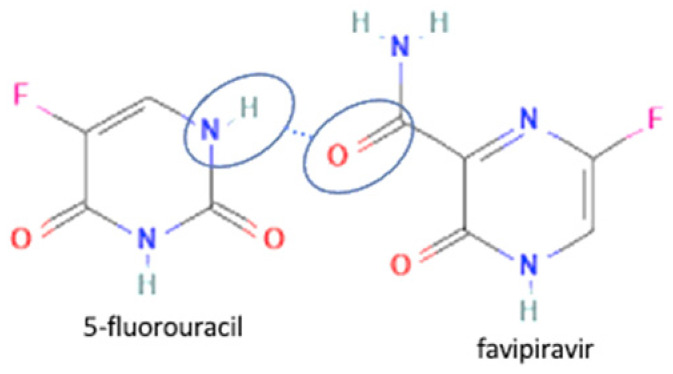	Enhanced the permeability and tablet ability of favipiravir	Liquid assisted grinding	[134]
71		Abacavir-azelaic acid	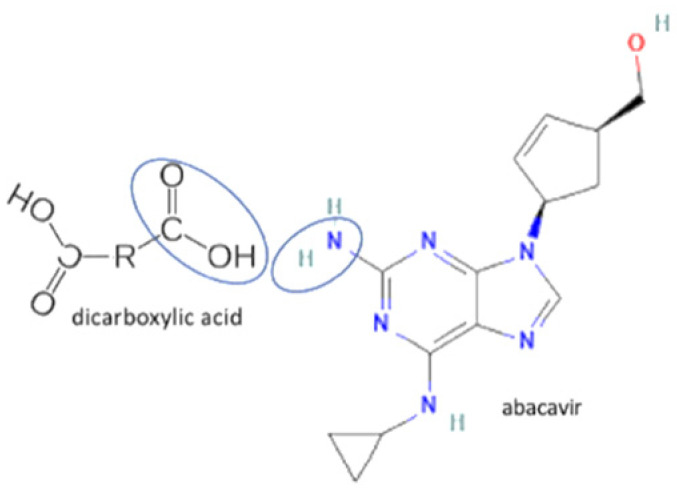	Enhancing the aqueous solubility and permeability rate of abacavir	Solvent evaporation	[135]
72		Abacavir-suberic acid		Enhancing the aqueous solubility and permeability rate of abacavir	Solvent evaporation	[135]
73		Amantadine-sulfamethoxazole	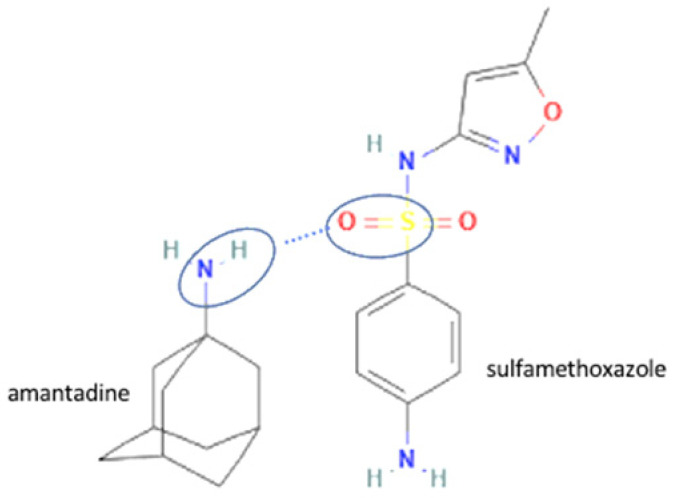	Permeability and dissolution improvement over the bulk drug and the bacterial activity of sulfamethoxazole are getting stronger.	Liquid-assisted grinding and solvent evaporation	[148]
74	Increasing powder properties	Efavirenz-fumaric acid	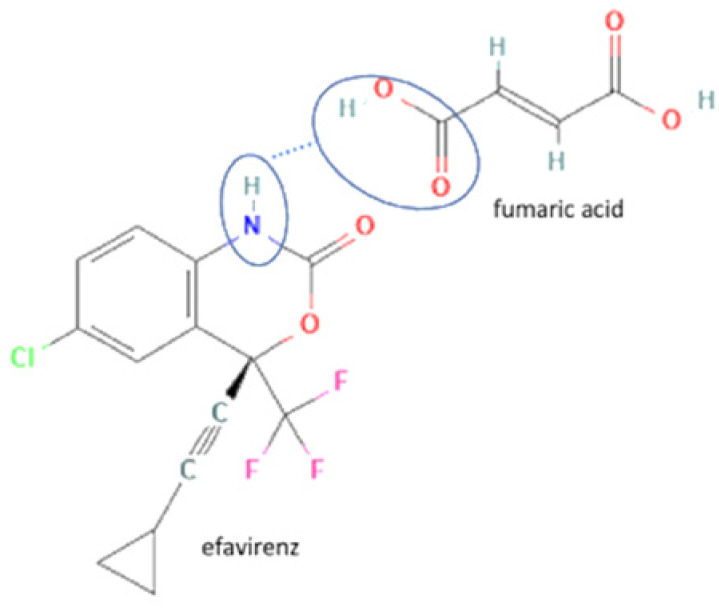	Increasing the powder flow properties, solubility, and dissolution profile of efavirenz.	Neat grinding	[149]
75	Decreasing hygroscopicity	Penciclovir-4 hydroxycinnamic acid	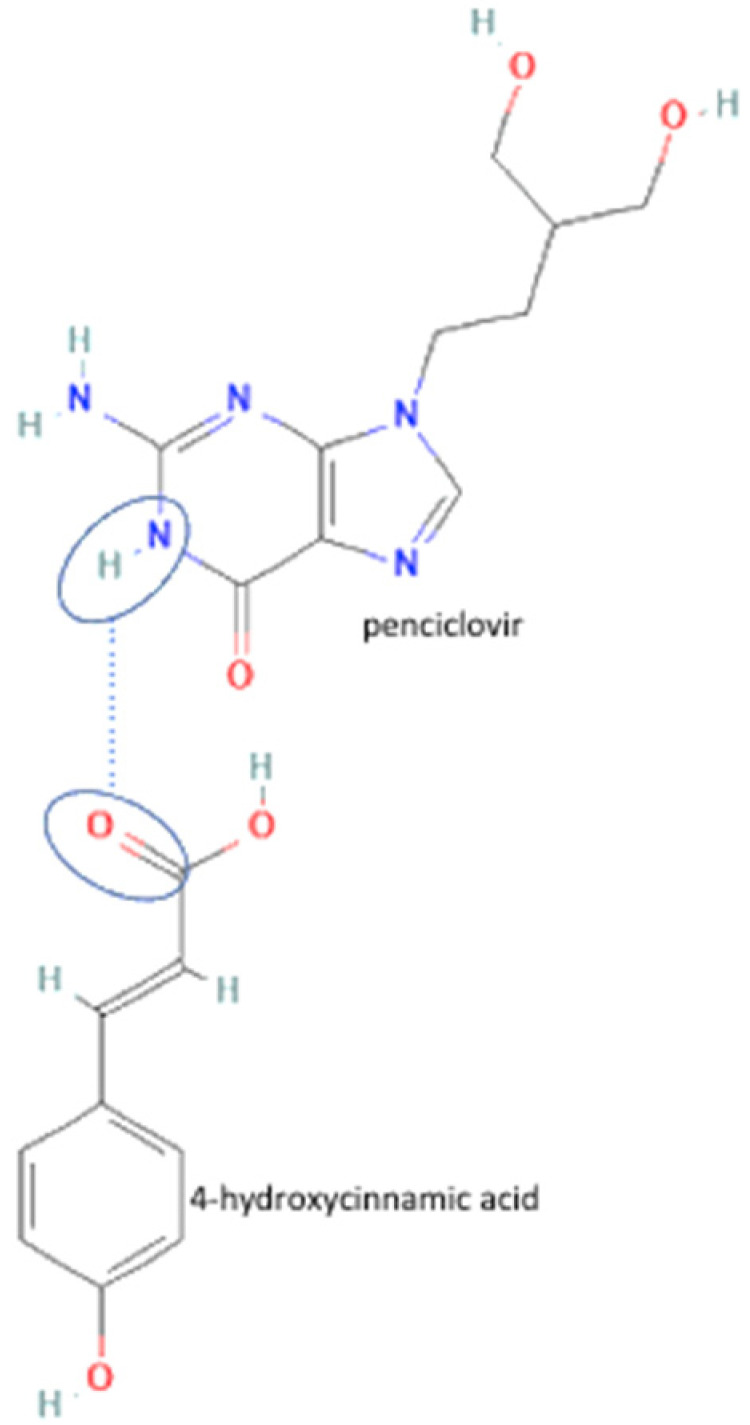	Decreasing hygroscopicity of penciclovir	Liquid-assisted grinding and slurry	[55]
76		Adefovir-maleic acid	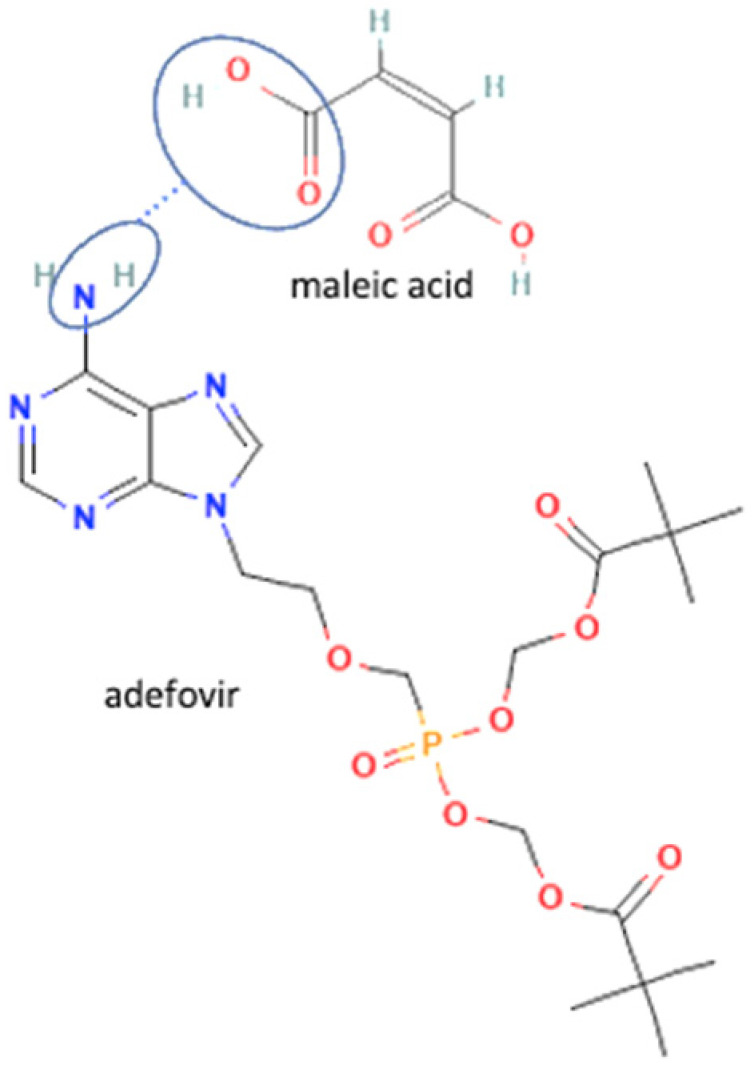	Decreasing hydration of adefovir	Slow evaporation	[37]
77	Crystal energy improvement	Arbidol-salicylic acid-CHCl3	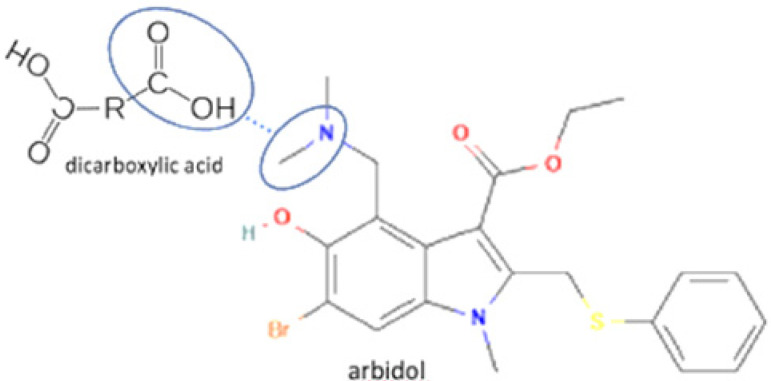	Lowering crystal energy to gain superior crystal energy, stronger solvent bond	Cooling cocrystallization	[40]
78		Arbidol-maleic acid		Lowering crystal energy to gain superior crystal energy, stronger solvent bond	Cooling cocrystallization	[40]
79		Arbidol-gallic acid		Lowering crystal energy to gain superior crystal energy, stronger solvent bond	Cooling cocrystallization	[40]

Based on the reported research in these past ten years, the most significant advantage of multicomponent formation is solubility enhancement, which increases the dissolution rate. This physicochemical property is one of the crucial parameters in pharmaceutical regulation. It affects the drug solubilization in the body and its bioavailability. Most of the antivirals have poor solubility in water, so the multicomponent system was formed, which proposes enhancing the antivirals’ solubility. The antivirals are commonly combined with the amino acids or carboxylic acids group to gain superior solubility. They are used as a coformer since they have many hydrogen donors where the sites form hydrogen bonds with the antivirals. They also have good solubility in water since weak acids easily dissociate. For example, favipiravir was combined with the gallic acid using liquid-assisted grinding, increasing the favipiravir solubility in buffer pH 7 into 20 folds, leading to dissolution rate enhancement [142]. Abacavir and oxalic acid multicomponent have increased the abacavir solubility by two-fold. Furthermore, it also affects the permeability of the abacavir [135]. Meanwhile, the nevirapine and fumaric acid multicomponent has improved the nevirapine solubility but lowered the multicomponent’s melting point, leading to decreased thermal stability [83].

Besides solubility, antiviral stability can be improved by multicomponent formation. Stability is a critical physicochemical property affecting the safety and efficacy of the drug’s administration. For example, the thermodynamical stability of adefovir dipivoxil was significantly enhanced by the multicomponent compound formation with saccharin. It was tested at 60 °C and resulted in no degradation compound in the multicomponent, but the antiviral alone contains impurities. Adefovir-dipivoxil is unstable since the high temperature can easily damage the P=O group, which is degraded into mono-POM and PMEA. In the adefovir-saccharin multicomponent, P=O of adefovir was linked up with the NH group in saccharin by hydrogen bonding and prevented the adefovir degradation in high temperatures [37], the interaction between adefovir and saccharin can be seen in Table 2 (No. 56). The multicomponent system stability significantly impacts manufacturing and storage, leading to a longer shelf-life. In addition, it can also improve the powder property, which leads to better manufacturing and formulation. Efavirenz-fumaric acid is an antiviral multicomponent system that improved the powder flow properties of efavirenz. It is prepared using a neat grinding method [149].

Lastly, multicomponent development has also been reported to improve antiviral activity by a synergistic effect. For example, the popular combination of lamivudine-favipiravir has been a potent antiviral in HIV treatment for almost a decade. Next, amantadine hydrochloride has been combined with ferulic acid, enhancing antiviral activity. The multicomponent formation was formed by charged-assisted hydrogen bonds containing chloride ions, which crucially maintained the crystal lattice of the multicomponent. This multicomponent showed an antiviral synergistic effect of amantadine hydrochloride and ferulic acid with a combination index (CI) of less than 1 [147]. This improvement confirmed that the multicomponent system did not change the drug’s efficacy since it only changed the physical structure but not the chemical structure. Moreover, the amantadine hydrochloride-resveratrol multicomponent has also enhanced the antiviral activity of amantadine hydrochloride. A dye uptake assay was conducted in the antiviral assay; samples were dissolved in DMSO and tested in the cells infected by various viruses.

The antiviral activity should be tested against several viral strains. The inhibition concentration/IC50 value can be used to determine the antiviral activity. The lower value indicates good antiviral activity. Amantadine hydrochloride-resveratrol multicomponent has significantly lower IC50 values (*p* < 0.05), and the synergistic effect is shown by the combination index (CI) value of less than 1. This synergistic effect occurred since both compounds have antiviral activity. Amantadine hydrochloride is an M2 ion channel inhibitor, and resveratrol can inhibit the expression of neuraminidase in the antiviral process. Therefore, different action targets may lead to synergistic effects and overcome the multitarget antiviral effects [147].

## 5. Conclusions

Solid engineering has been a practical approach to improving drug performance and activity, including antiviral multicomponent systems arrangement for decades. In addition, solid analysis instruments have also been developed to support this development. As a result, many combinations of antivirals with other drugs, nutraceuticals, and excipients have been reported to possibly improve solubility, stability, powder properties, and activity. Hereafter, timely, the multicomponent system arrangement is expected to be one of the best strategies to be used in antiviral development.

## Figures and Tables

**Table 1:** Summary of Antiviral Mechanism and Its Indication.

No.	Mechanisms	Antiviral	Indication
1.	Nucleoside Reverse Transcriptase Inhibitor (NRTI) Block the synthesis of viral nucleic acid	Acyclovir	- Oral First episode genital herpes treatment, recurrent genital herpes treatment, genital herpes suppression, herpes proctitis treatment, first episode orolabial herpes treatment, recurrent orolabial herpes treatment, orolabial herpes suppression, varicella treatment (age ≥ 2 years), zoster treatment - Intravenous Severe herpes simplex virus treatment, mucocutaneous herpes in the immunocompromised host treatment, herpes encephalitis treatment, neonatal herpes simplex virus infection treatment, varicella or zoster in the immunosuppressed host treatment - Topical (5% cream) Herpes labialis treatment
		Ganciclovir	- Topical (0.15% gel) Keratitis - Intravenous cytomegalovirus retinitis treatment
		Valganciclovir	Oral cytomegalovirus retinitis treatment, cytomegalovirus prophylaxis (transplant patients)
		Penciclovir	Topical (1% cream) Herpes labialis or herpes genitalis
		Valacyclovir	Oral First episode genital herpes treatment, recurrent genital herpes treatment, genital herpes suppression, first episode orolabial herpes treatment, recurrent orolabial herpes treatment, orolabial herpes treatment, varicella (age > 2 years), zoster
		Famciclovir	Oral First episode genital herpes treatment, recurrent genital herpes treatment, genital herpes in the HIV-infected host treatment, genital herpes suppression, first episode orolabial herpes treatment, recurrent orolabial herpes treatment, orolabial herpes suppression, zoster
		Cidofovir	Intravenous cytomegalovirus retinitis treatment
		Trifluridine	Topical (1% solution) Acyclovir-resistant herpes simplex virus infection
		Foscarnet	Intravenous Acyclovir-resistant herpes simplex virus and varicella-zoster virus infection, cytomegalovirus retinitis treatment
		Lamivudine	Oral Chronic hepatitis B, antiretroviral (in pregnancy)
		Zidovudine	First-line antiretroviral (in pregnancy), decrease the rate of clinical disease progression and prolong survival in HIV-infected individuals.
		Abacavir	Oral Antiretroviral (in pregnancy)
		Emtricitabine	Oral Antiretroviral (in pregnancy)
		Tenofovir	Oral Chronic hepatitis B HBV infection; antiretroviral (in pregnancy); pre-exposure prophylaxis to reduce HIV acquisition in men who have sex with men, in heterosexually active men and women, and in injection drug users.
		Stavudine	Oral Antiretroviral
		Didanosine	Antiretroviral
		adefovir dipivoxil	Oral Chronic hepatitis B
		Entecavir	Oral Chronic hepatitis B
		Telbivudine	Oral Chronic hepatitis B
2.	Protease Inhibitor (PI) Block viral late protein synthesis and processing	Ritonavir	Antiretroviral (in pregnancy)
		Saquinavir	Antiretroviral (in pregnancy)
		Tipranavir	Antiretroviral
		Atazanavir	Antiretroviral (in pregnancy)
		Lopinavir	Antiretroviral (in pregnancy)
		Darunavir	Antiretroviral (in pregnancy)
		Nelfinavir	Antiretroviral
		Indinavir	Antiretroviral
		Fosamprenavir	Antiretroviral
		Boceprevir	Oral Chronic hepatitis C
		Telaprevir	Oral Chronic hepatitis C
		Simeprevir	Oral Hepatitis C
3.	Nonnucleoside Reverse Transcriptase Inhibitor (NNRTI) Block the synthesis of viral nucleic acid	Nevirapine	Antiretroviral (in pregnancy)
		Rilpivirine	Antiretroviral
		Etravirine	Antiretroviral
		Efavirenz	Antiretroviral
		Delavirdine	Antiretroviral
4.	Integrase Strand Transfer Inhibitors (INSTI) Block viral nucleic acid integration into the genome	Raltegravir	Antiretroviral
		Elvitegravir	Antiretroviral
		Dolutegravir	Antiretroviral
5.	Entry Inhibitors Block viral attachment and entry into the cell	Maraviroc	Oral Treatment of experienced adult patients infected with only CCR5-tropic HIV-1 detectable who are resistant to other antiretroviral agents
		Enfuvirtide	Subcutaneous Antiretroviral (HIV)
		Docosanol	Topical (10% cream) Keratitis
6.	Interferons (speculated to have multiple sites of action)	interferon alfa 2b	Subcutaneous/intramuscular Chronic hepatitis B, acute hepatitis C
		pegylated interferon alfa 2a	Subcutaneous Chronic hepatitis B, chronic hepatitis C
		pegylated interferon alfa 2b	Subcutaneous Chronic hepatitis C
7.	Neuraminidase Inhibitor Block viral release from the cell	Oseltamivir	Oral Anti-influenza A and B
		Zanamivir	Inhalation Anti-influenza A and B
		Peramivir	Intravenous Anti-influenza A and B
8.	Inhibit viral uncoating process	Amantadine	Anti-influenza A
		Rimantadine	Anti-influenza A

## Data Availability

Not applicable.
